# The low-affinity complex of cytochrome *c* and its peroxidase

**DOI:** 10.1038/ncomms8073

**Published:** 2015-05-06

**Authors:** Karen Van de Water, Yann G. J. Sterckx, Alexander N. Volkov

**Affiliations:** 1 Jean Jeener NMR Centre, Structural Biology Brussels, Vrije Universiteit Brussel, Pleinlaan 2, Brussels 1050, Belgium; 2 Structural Biology Research Center, VIB, Pleinlaan 2, Brussels 1050, Belgium; 3 Research Unit for Cellular and Molecular Immunology (CMIM), Vrije Universiteit Brussel, Pleinlaan 2, Brussels 1050, Belgium

## Abstract

The complex of yeast cytochrome *c* peroxidase and cytochrome *c* is a paradigm of the biological electron transfer (ET). Building on seven decades of research, two different models have been proposed to explain its functional redox activity. One postulates that the intermolecular ET occurs only in the dominant, high-affinity protein–protein orientation, while the other posits formation of an additional, low-affinity complex, which is much more active than the dominant one. Unlike the high-affinity interaction—extensively studied by X-ray crystallography and NMR spectroscopy—until now the binding of cytochrome *c* to the low-affinity site has not been observed directly, but inferred mainly from kinetics experiments. Here we report the structure of this elusive, weak protein complex and show that it consists of a dominant, inactive bound species and an ensemble of minor, ET-competent protein–protein orientations, which summarily account for the experimentally determined value of the ET rate constant.

## Abstract (web summary)

The redox activity of cytochrome *c* in complex with its peroxidase has been rationalized by two possible models; a readily observable high-affinity complex and a more elusive but potentially more active low-affinity complex. Here, the authors provide an NMR-based structural mapping of this low-affinity complex.

## Graphical abstract

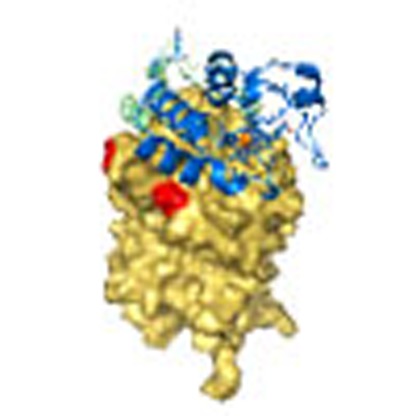

Biological electron transfer (ET) reactions are essential for many vital cellular activities including oxidative phosphorylation and photosynthesis—two processes underlying the conversion of energy from food or sunlight into the chemical energy of adenosine triphosphate. The complex of yeast proteins cytochrome *c* (Cc) and cytochrome *c* peroxidase (CcP) is the paradigm for the study of biological ET. Located in yeast mitochondria, CcP is a haem enzyme, which reduces hydroperoxides using the electrons provided by its physiological partner Cc. The catalytic mechanism of H_2_O_2_ reduction involves the formation of CcP compound I (CpdI), an intermediate oxidized two equivalents above the CcP(Fe^3+^) resting state and containing the Fe(IV)=O haem oxyferryl and the W191^+·^ cation radical^1^,^2^. Subsequent CpdI reduction in two one-electron steps involves complex formation with ferrous Cc, intermolecular ET and the product dissociation. On the basis of a substantial body of experimental work (reviewed in refs 1, 2), two contrasting models have been put forward to explain the ET activity. According to the first one^3^,^4^,^5^,^6^, the ET occurs only from the Cc bound at the high-affinity site of CcP as seen in the X-ray structure of the complex^7^. An alternative mechanism proposes the existence of multiple ET-active protein–protein orientations and postulates that the ET from the Cc bound to a low-affinity site is faster than that in the crystallographic orientation^8^,^9^,^10^,^11^. Since then, the structure and ET properties of the low-affinity Cc–CcP complex have been a matter of active interest and ongoing debate.

The first conclusive evidence for the 2:1 Cc–CcP complex formation came from the studies of Hoffman and co-workers^8^,^9^,^10^, who followed triplet-state quenching of Zn-substituted CcP or Cc by varying concentrations of the respective redox partner. The observed, rather complex, kinetics were explained by a model postulating two non-interacting Cc-binding sites of markedly different affinities and ET properties. Because of the big (*∼*10,000-fold) difference in the Cc affinities for the two binding sites, biophysical characterization of the ternary complex turned out to be an exceedingly difficult task, requiring a highly sensitive technique with a large dynamic range to detect small binding effects at the low-affinity site in the presence of a much stronger, dominant signal from the high-affinity site. To the best of our knowledge, only two non-kinetic, equilibrium studies have presented strong evidence for formation of the 2:1 Cc–CcP complex. In their early potentiometric work, Mauk *et al.*^12^ observed Cc binding to two CcP sites with different protonation properties and established that the ternary complex is salt sensitive, with the second binding event abolished at high ionic strength (*I*≥100 mM). Then, in a more recent calorimetry study, Morar and Pielak^13^ reported the 2:1 binding in the Cc–CcP system stabilized by trisaccharide melezitose and showed that the CcP mutation D148A disrupts the ternary complex formation, suggesting that this residue might be part of the low-affinity site. Despite these efforts, the exact location of the low-affinity site remains unclear, its binding properties in the absence of the sugar uncertain and the structure of the low-affinity complex unknown.

To resolve these unknowns, here we present a structural characterization of the elusive low-affinity complex in solution. Two crucial elements were integral to the success of this study—decoupling of the binding events at the two sites and the use of solution NMR spectroscopy, in particular paramagnetic NMR, to obtain the relevant structural information. To achieve the first goal, we blocked the high-affinity CcP site by crosslinking (CL) the partner proteins via an intermolecular disulfide bond. With an overall positional root mean squared deviation of 2.5 Å between C_α_ atoms of the CcP(V197C)-Cc(A81C) product and the native Cc–CcP complex^14^, this site-specific, covalent CL is an excellent structural mimic of the high-affinity binding geometry. Thus, with the proteins locked in the nearly-native crystallographic orientation, this CL enables the study of the low-affinity Cc–CcP interaction in the absence of strong binding effects from the high-affinity site.

To solve the structure of the CL–Cc complex, we used paramagnetic relaxation enhancement (PRE) NMR spectroscopy, a powerful technique for studying weak complexes of soluble proteins^15^,^16^,^17^,^18^ and characterizing minor states in the interactions of membrane-associated and integral membrane proteins^19^,^20^. The PRE is caused by a dipolar interaction between a nucleus and the unpaired electron(s) of the paramagnetic center, either present in the native protein or introduced by bioconjugation techniques. Because of the large magnetic moment of an unpaired electron and the *r*^−6^ distance dependence, the PRE effect is long-range (extending up to 35 Å) and exquisitely sensitive to lowly populated species^21^,^22^. Manifested by the decrease in NMR signal intensities, and generally measured as differences in the transverse relaxation rates of protons in the paramagnetic sample and a diamagnetic reference, ^1^H Γ_2_ PREs can be used as restraints in subsequent structure calculations^21^,^22^.

Here we present a structural analysis of the low-affinity Cc–CcP complex in solution and show that it consists of a dominant protein–protein orientation and an ensemble of minor binding geometries, populating electrostatically favourable regions of the interaction space. We obtain individual, microscopic binding constants for the two species and assess their ET properties. Judging from the large distances between the Cc and CcP redox centres, the dominant binding form is inactive in the intermolecular ET. At the same time, the conformational ensemble constituting the minor species contains multiple, ET-competent protein–protein orientations with short haem–haem separations. The calculated ET rate constant is in excellent agreement with the experimentally measured value, suggesting that the low-affinity complex accounts for the measured haem–haem ET activity. Our findings settle the long-standing debate on the nature of the low-affinity Cc–CcP interaction and provide mechanistic insights into the intermolecular ET process in this paradigmatic system.

## Results

### Synthesis and characterization of the disulfide CLs

**Figure 1:**
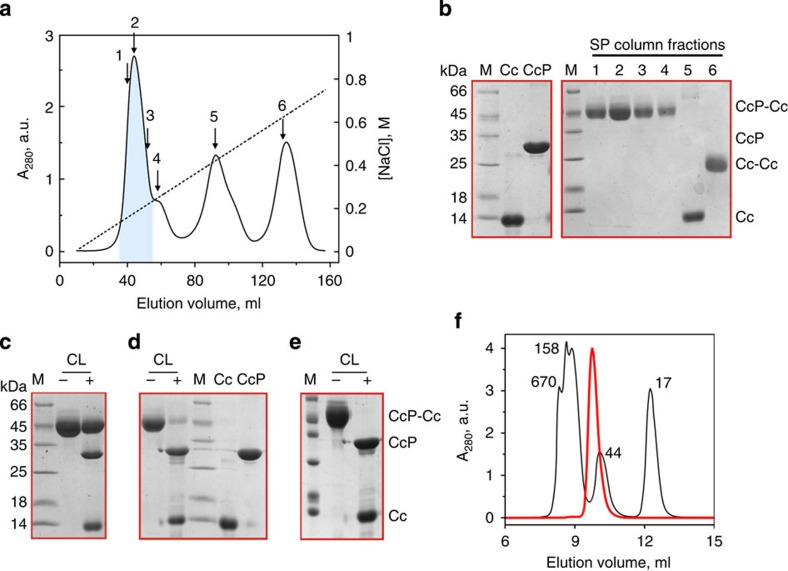
Characterization of the [D, ^15^N] V197C CcP–A81C Cc disulfide CL. (**a**) Purification of the CL by cation-exchange chromatography. The solid and dashed lines show the ultraviolet absorbance and the linear salt gradient (from 0 to 1 M NaCl in 20 mM NaP_i_ pH 6.0), respectively, for protein elution from a HiTrap SP FF column (GE Healthcare). Protein fractions analysed by SDS–PAGE are indicated by the arrows. The pooled fractions, containing the pure CL, are highlighted. (**b**) Non-reducing SDS–PAGE of reference samples (wt proteins) and the SP column fractions labelled in **a**. (**c**–**e**) SDS–PAGE with (+) and without (−) the disulfide reducing agent DTT of (**c**,**d**) the freshly purified CL and (**e**) the CL stock used for NMR experiments and stored for 1 month at 4 °C. Incubated for 15 min at room temperature, the ‘+' sample in (**c**) contained 10 mM DTT, while those in (**d**,**e**) contained 125 mM DTT and were incubated for 15 min at 99 °C before analysis. For reference, the wt protein samples are included in **d**. In **b**–**e**, ‘M' denotes the molecular weight marker, with the values indicated on the left. (**f**) Analysis of the purified CL by analytical size-exclusion chromatography (SEC). Black and red lines show elution of the SEC protein standard (Bio-Rad) and the CL sample, respectively, from ENrich SEC 70 column (Bio-Rad) equilibrated in 20 mM NaP_i_ 0.1 M NaCl pH 6.0. The molecular weights (in kDa) of the reference proteins are indicated in the chromatogram. The expected molecular weight of the CL is 46 kDa.

To prepare covalent, disulfide-linked Cc–CcP complexes, we adopted the experimental strategy of Otting and co-workers^23^, wherein one of the single-cysteine protein variants is activated by Ellman's reagent and then reacted with the free thiol-bearing binding partner (see Methods). Purified from the reaction mixture by ion-exchange chromatography, the resulting covalent CL elutes in a single peak, well separated from those of other protein components (Fig. 1a). To ensure the highest purity of the final sample, only the central part of the CL elution peak was collected (highlighted in Fig. 1a), leaving out protein fractions at the leading and trailing edges. Estimated by ultraviolet–visible spectrophotometry of the final [D, ^15^N] V197C CcP–A81C Cc CL stock solution, the overall yield of CL was at least 85 %. (The actual value is expected to be higher as not all CL fractions were included in the analysis.) The CL yield achieved here is substantially higher than 25–40% reported for the original CL experiments of Pappa and Poulos^24^ or >60 % estimated in their follow-up work^14^, both of which employed Cu^2+^-catalysed oxidation of protein thiols to promote the intermolecular disulfide formation.

As shown in Fig. 1b–e, the [D, ^15^N] V197C CcP–A81C Cc CL is highly pure and appears to be remarkably stable. In agreement with an earlier report^14^, treatment of this CL with 10 mM dithiothreitol (DTT) fails to reduce the intermolecular disulfide (Fig. 1c), which can be achieved only under strongly denaturing conditions with large amounts of the reducing agent (Fig. 1d). Stored at 4 °C, the CL stock solution remains stable for more than a month (Fig. 1e), a period of time largely sufficient for the NMR experiments conducted in this work.

The two-dimensional (2D) [^1^H, ^15^N] transverse relaxation-optimized spectroscopy (TROSY) spectrum of [D, ^15^N] V197C CcP(CN)–A81C Cc CL is highly similar to that of the free [D, ^15^N] wild-type (wt) CcP(CN)^25^, enabling a facile transfer of backbone amide resonance assignments. The NMR signals of CcP residues 22–30 and 99–119, which are already weak in the free protein^25^, disappear in the CL, most likely because of a faster transverse relaxation caused by an increased rotational correlation time of the heavier CcP–Cc CL. In addition, the resonances of the CcP CL residues 193–199, located at the intermolecular interface, either disappear or shift to new spectral positions, which cannot be unambiguously assigned from an overlay of the two [^1^H, ^15^N] TROSY spectra. Overall, the high similarity of the NMR spectra of the free and Cc-linked CcP confirms that CL does not perturb the CcP molecule^14^ and suggests that the individual proteins maintain their original structures, which justifies the rigid-body refinement of Cc–CcP complexes used in this and earlier works^26^,^27^,^28^.

Similarly to the V197C CcP–A81C Cc CL discussed above, the other CLs used in this work can be obtained in high yield and purified to homogeneity (Supplementary Figs 1 and 2). As established by SDS–PAGE (polyacrylamide gel electrophoresis) analysis, the final products correspond to the desired CLs. Represented by the sum of absorptions of individual proteins^29^, the ultraviolet–visible spectrum of a covalent complex (Supplementary Fig. 1b) confirms its purity and indicates that 3-thio-6-nitorbenzoate (TNB), used to activate cysteine residues during the CL reaction and featuring strong absorbance at 412 nm (ref. 30) does not co-purify with the protein samples.

### Chemical shift perturbation analysis

**Figure 2:**
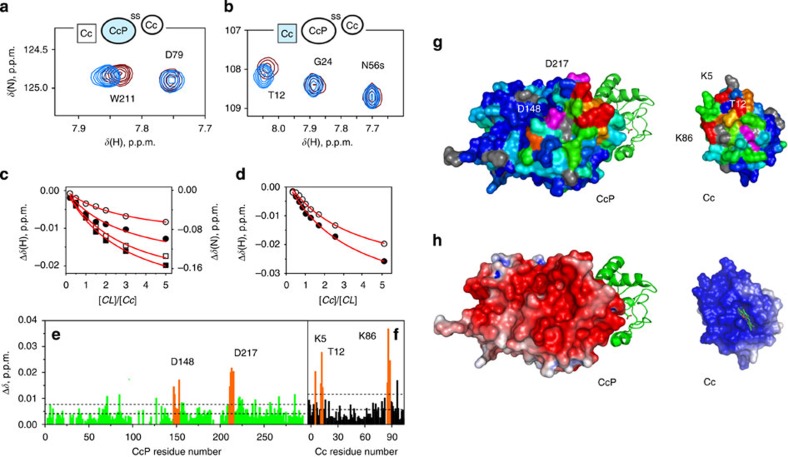
Binding analysis of the low-affinity Cc–CcP complex. The CcP-observed Cc binding to the V197C CcP–A81C Cc CL and the Cc-observed binding of the CL in 20 mM NaP_i_ pH 6.0 at 25 °C. (**a**,**b**) Selected regions of the overlaid [^1^H, ^15^N] heteronuclear single-quantum correlation spectra of the free proteins (blue) and in the presence of 5 equivalents of the corresponding binding partner (brown), showing typical chemical shift perturbations upon complex formation. The insets schematically depict the experimental set-up, with the NMR-active proteins coloured light blue. The symbol ‘s' identifies a sidechain amide resonance. (**c**,**d**) NMR chemical shift titrations of (**c**) CL CcP nuclei A147 HN (open circles), D148 HN (filled circles), E214 HN (open squares) and L213 N (filled squares) and (**d**) Cc HN atoms of K86 (filled circles) and K5 (open circles). The curves in each plot were fitted simultaneously to a binding model with the shared *K*_d_. The solid lines show the best fits with the *K*_d_ values of 1.74±0.25 mM (CL-observed) and 2.30±0.66 mM (Cc-observed). (**e**,**f**) Binding-induced, combined chemical shift perturbations (Δ*δ*) of the backbone amides of (**e**) CL CcP and (**f**) Cc. The horizontal lines indicate the average Δ*δ* and the average plus one standard deviation. Several clusters of residues most affected by the binding are indicated by the labels, with the Δ*δ* coloured orange. (**g**) The Δ*δ* heat maps (coloured from 0.001 p.p.m. in blue to 0.02 p.p.m. in red; prolines and the residues with unassigned or unobserved backbone amide resonances are in grey) for the CL CcP and Cc. The labels indicate several residues affected by the binding. (**h**) Electrostatic properties of Cc and CcP, with molecular surfaces coloured by the electrostatic potential (from −5 *k*_B_*T* in red to +5 *k*_B_*T* in blue) calculated with APBS^57^. The molecular views in **g**,**h** show X-ray structure of the Cc–CcP CL (PDB 1S6V)^14^ with Cc as the green ribbon and CcP as the molecular surface and the Cc interface, with the haem group (**g**) coloured magenta or (**h**) shown in sticks.

Having prepared the protein CLs, we performed NMR chemical shift perturbation (Δ*δ*) analysis of the CL–Cc interaction (Fig. 2). To observe binding effects on both proteins, we monitored (1) the Cc complex formation with [D, ^15^N] isotopically labelled, NMR-active CcP cross-linked to the natural-abundance, NMR-silent Cc and (2) binding of the NMR-active, ^15^N-labelled Cc to the Cc–CcP CL in which both proteins are NMR-silent (Fig. 2a,b, respectively). As seen in the [^1^H, ^15^N] correlation spectra of the ^15^N-labelled proteins (Fig. 2a,b), stepwise addition of the binding partner leads to incremental shifts for several backbone amide resonances, indicating that the CL–Cc interaction is in fast exchange. Analysis of the CL- and Cc-observed NMR titration curves provided very similar values for the equilibrium dissociation constant (*K*_d_), suggesting that the effects observed from both protein sides report on the same binding event (Fig. 2c,d). The combined *K*_d_ value of 2.0±0.4 mM obtained in this work is in good agreement with an earlier estimate, *K*_d_≥1 mM, for the low-affinity binding under similar experimental conditions^12^.

As seen in other NMR studies of such weak protein–protein interactions^17^,^18^,^31^, the chemical shift perturbations observed here are small (Fig. 2a,b,e,f), reflecting the modest equilibrium population of the Cc–CL complex in the NMR samples. The Δ*δ* analysis reveals that, for both proteins, the binding effects are confined to several clusters of residues constituting contiguous surface patches (Fig. 2g). Most of the affected CcP groups are found in the region bordering D148 and D217, identified in a classic Brownian dynamics study as the likely location of the low-affinity site^32^. On the Cc side, the binding interface is defined by K5, T12 and K86, encompassing an area adjacent to the exposed haem edge. Believed to be driven by complementary electrostatics, the low-affinity Cc–CcP interaction indeed appears to involve oppositely charged protein surfaces as demonstrated by the present NMR analysis and Poisson–Boltzmann calculations (Fig. 2g,h). Finally, our conclusions are reinforced by the control experiments showing that the Cc binding to CcP(E290C)-Cc(K73C) CL, an alternative mimic of the high-affinity complex, proceeds via essentially the same protein interface and with the same *K*_d_ as those of the CL discussed above (Supplementary Fig. 3).

### PRE NMR spectroscopy

**Figure 3:**
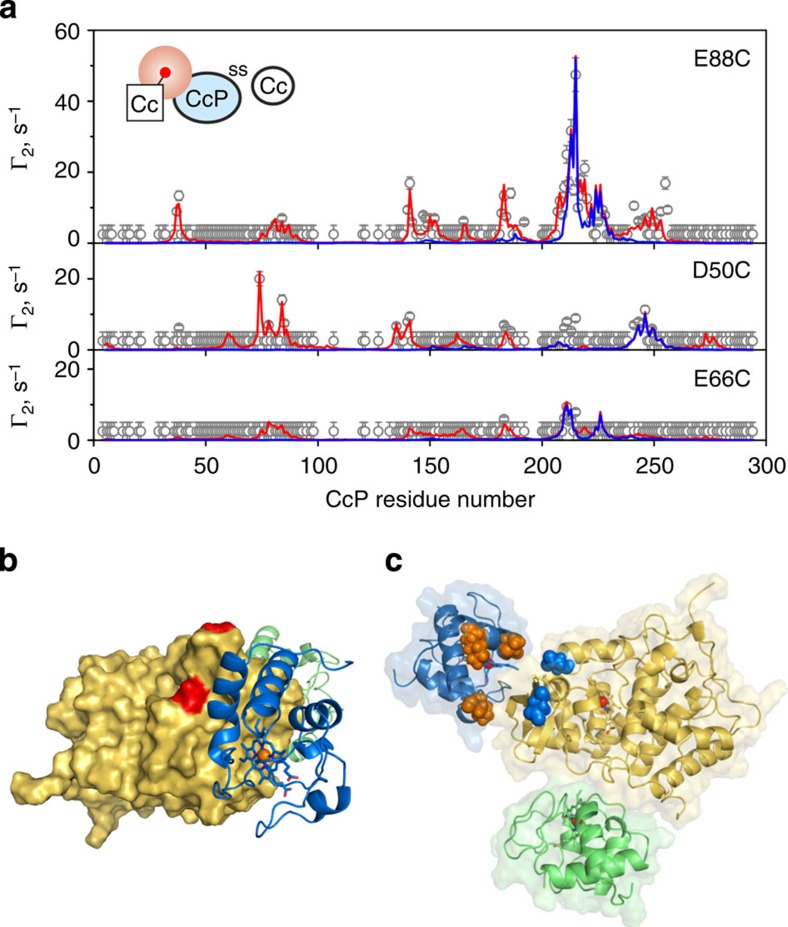
Structure of the low-affinity Cc–CcP complex. (**a**) Intermolecular, CcP-observed PREs for the CL in the complex with Cc paramagnetically labelled at E88C, D50C, and E66C. The plots show measured PREs (open symbols), Γ_2_ values back-calculated from the single, lowest-energy CL–Cc structure (blue line) and the PREs calculated for the combination of the dominant binding geometry and multiple protein–protein orientations obtained in a typical ensemble refinement run (red line). The errors are s.d. The inset schematically depicts the experimental set-up, with the NMR-active protein coloured light blue and the attached paramagnetic label indicated by the red sphere. (**b**) The structure of the dominant form of the CL–Cc complex. Cc bound to the low-affinity CcP site is shown as blue cartoon, while the CL CcP and Cc are represented as the yellow molecular surface and the green cartoon, respectively. The CL orientation is the same as in Fig. 2g. CcP residues D148 and D217 are coloured red. Haem groups are shown as sticks, with iron atoms as spheres. (**c**) The intermolecular interface of the dominant low-affinity binding orientation. The proteins are coloured as in **b**. CcP residues D148 and D217 and Cc residues K5, T12 and K86 spacefilled and shown in blue and orange, respectively.

The Δ*δ* analysis provides good qualitative description of the Cc–CL binding interface, yet it is insufficient for establishing the mutual orientation of the binding partners, that is, the structure of the complex^33^. To obtain the quantitative structural information necessary to achieve this goal, we used PRE NMR spectroscopy. Here intermolecular PREs for the low-affinity Cc–CcP complex were obtained in essentially the same experimental set-up as that used in our recent study of the high-affinity Cc–CcP interaction^28^. In particular, three single-cysteine Cc variants D50C, E66C and E88C were labelled with an EDTA-based, chelating tag containing a paramagnetic metal ion (Mn^2+^, *S*=5/2), and their interactions with [D, ^15^N] CcP cross-linked to the natural-abundance Cc were monitored in [^1^H, ^15^N] TROSY experiments. Following an established methodology^28^, a set of intermolecular PREs—detected on the CcP backbone amide protons—was obtained from a series of TROSY spectra, yielding distinct Γ_2_ profiles for each Cc-EDTA(Mn^2+^) variant (Fig. 3a). Driven by the combined set of Γ_2_ restraints from all three EDTA(Mn^2+^) positions, structure calculations of the Cc–CL complex produced a set of well-defined low-energy solutions (Fig. 3b and Supplementary Fig. 4). Featuring Cc bound to the CcP face bordered by D148 and D217 residues, the low-affinity Cc–CcP-binding geometry is in excellent agreement with the chemical shift perturbation maps (Fig. 3c, also compare Figs 3b and 2g). This finding is even more striking as no Δ*δ* data were used in the molecular refinement. With the shortest distance of 19 Å between any two heavy atoms of the two cytochromes, there is no direct interaction between Cc molecules bound to the low- and high-affinity sites.

As can be seen in Fig. 3a, not all Γ_2_ restraints are accounted for by the single Cc–CL structure. Observed before for the high-affinity Cc–CcP complex^26^,^28^—shown to comprise the dominant binding form and an ensemble of lowly populated protein–protein orientations sampled in the transient encounter state—such additional PREs are the footprint of minor species^21^,^22^. Unlike the chemical shift perturbation, which is a linear, population-weighted average of Δ*δ*s in different protein–protein orientations, PRE is an <*r*^−6^ >-dependent effect. Thus, if the electron–nucleus distance in the minor form is shorter than that in the dominant one, the former will give rise to a very large PRE, making a measurable contribution to the overall, population-weighted *Γ*_2_ value^21^,^22^. This explains why additional effects, not present in the Δ*δ* plots, are observed in the Γ_2_ profiles.

**Figure 4:**
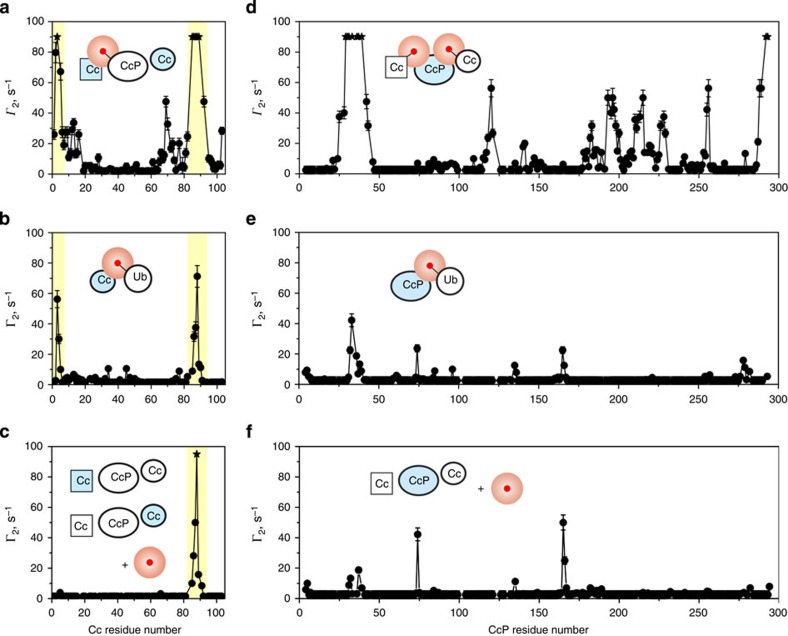
PRE control experiments. Intermolecular PREs for the backbone amide resonances of (**a**–**c**) ^15^N Cc caused by (**a**) CcP E221C-EDTA(Mn^2+^), (**b**) Ub D32C-EDTA(Mn^2+^) and (**c**) free paramagnetic label; and (**d**–**f**) [D, ^15^N] wt CcP caused by (**d**) Cc E88C-EDTA(Mn^2+^), (**e**) Ub D32C-EDTA(Mn^2+^) and (**f**) free paramagnetic label. Stars indicate the residues whose resonances disappear in the paramagnetic spectrum. The errors are s.d. The insets schematically depict the experimental set-up, with the NMR-active protein coloured light blue and the paramagnetic label indicated by the red sphere. The Cc regions that experience strong PREs are highlighted. The NMR samples contained (**a**) 0.3 mM of CcP E221C-EDTA(Mn^2+^) and 3 equivalents of ^15^N Cc; (**b**) 0.3 mM ^15^N Cc and 3 equivalents of Ub D32C-EDTA(Mn^2+^); (**c**) 0.3 mM wt CcP, 0.45 mM each of ^15^N-labelled and natural-abundance Cc, and 0.45 mM of the free paramagnetic label; (**d**) 0.4 mM [D, ^15^N] wt CcP and 3 equivalents of Cc E88C-EDTA(Mn^2+^); (**e**) 0.4 mM [D, ^15^N] wt CcP and 3 equivalents of Ub D32C-EDTA(Mn^2+^). The sample in **f** was generated from that in **d** by addition of twofold molar excess (relative to Cc) of DTT, which breaks the disulfide bond between Cc and the EDTA(Mn^2+^) moiety, releasing the latter into the solution. All experiments were conducted in 20 mM NaP_i_ pH 6.0 at 25 °C.

As described in detail in our recent study of the high-affinity Cc–CcP complex, the EDTA(Mn^2+^) conjugation to Cc yields a single, well-defined product and does not perturb the native Cc–CcP binding^28^. To ascertain that the PREs presented in Fig. 3a are not experimental artifacts, but indeed arise from the Cc–CL interaction, we performed a number of control experiments. First, we addressed a possible aspecific binding of the attached paramagnetic probe to the NMR-active protein, which is of particular concern under the low ionic strength conditions of the present study. As shown in Fig. 4a, the Cc interaction with paramagnetically labelled CcP gives rise to very strong PRE effects, affecting two protein regions (highlighted). Control experiments with the paramagnetically labelled ubiquitin (Ub)—an unrelated protein that binds neither Cc nor CcP—yield Γ_2_ profiles with strong effects in the same two regions (Fig. 4b). Finally, Cc appears to bind the free EDTA(Mn^2+^) probe as evidenced by large PREs affecting one of the aforementioned protein areas (Fig. 4c). Taken together, these findings reveal an undesired interaction between oppositely charged Cc and the CcP-tethered EDTA(Mn^2+^) moiety, which precluded the Cc-observed NMR experiments with the paramagnetically labelled CcP, initially intended as part of this work.

Unlike in the Cc case, the CcP-observed control experiments testify to the absence of significant aspecific interactions (Fig. 4d–f). The wt CcP binding to Cc-EDTA(Mn^2+^) produces a Γ_2_ profile featuring large PREs, most of which arise from the high-affinity Cc–CcP binding form and the associated encounter complex (Fig. 4d)^28^. At the same time, experiments with paramagnetically labelled Ub and the free EDTA(Mn^2+^) show only a few weak effects (Fig. 4e, f), which are not found in the PRE profiles of either wt CcP or the CcP–Cc CL (Figs 4d and 3a, respectively). Moreover, distinct CcP-detected PRE patterns are observed for CL complexes with different Cc-EDTA(Mn^2+^) variants (Fig. 3a), which would be unlikely if the measured effects were dominated by the binding of the paramagnetic probe—expected to be largely insensitive to the attachment site. Finally, the intermolecular PREs observed in the CL–Cc system are reproducible (Supplementary Fig. 5), and the CL–Cc Γ_2_ effects are also detected in the native, non-covalent wt CcP–Cc complex (see below). These findings confirm that the measured PREs report on the Cc–CcP binding, and not a trivial protein-probe interaction.

### Structural and thermodynamic analysis of the minor species

**Figure 5:**
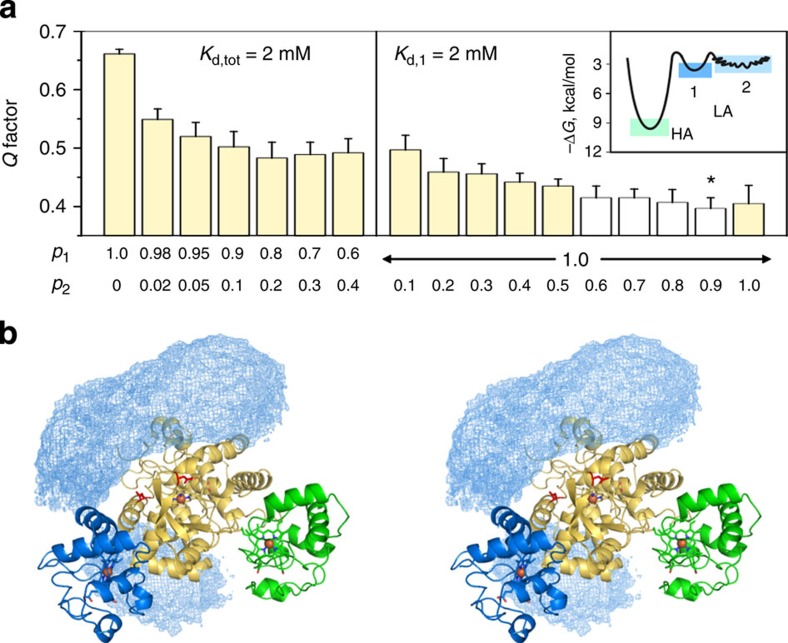
Ensemble refinement of the lowly populated CL–Cc forms. (**a**) *Q* factors for ensemble refinement of CL–Cc complexes at varying relative populations of the two bound species, *p*_1_ and *p*_2_, and two binding scenarios (see text). White bars indicate the smallest *Q* values. An asterisk identifies the data set presented in **b**. The inset shows a schematic energy diagram for the native, non-covalent Cc–CcP complex, where ‘HA' and ‘LA' refer to the high- and low-affinity domains, respectively, and ‘1' and ‘2' indicate the two binding sites of the latter (see text for details). (**b**) Stereo image showing Cc molecules in the dominant binding geometry (blue cartoon) and multiple minor forms of the low-affinity Cc–CcP complex, displayed as a reweighted atomic probability density map for the overall distribution of the Cc heavy atoms plotted at a threshold of 40% maximum^58^. The CL CcP and Cc are coloured yellow and green, respectively.

To estimate the population of the minor species and visualize the constituent protein–protein orientations, we performed conjoint refinement^34^ of the dominant Cc–CL complex and an ensemble of additional binding forms against the observed PREs. In practice, the relative populations of the two bound species, *p*_1_ and *p*_2_, were varied in multiple refinement runs. Each time, the agreement between the experimental data and the Γ_2_ values back-predicted from the resulting solutions was evaluated by calculating the *Q* factor (equation 1)—a quantitative measure of the fit, with smaller *Q* values indicating a better match^34^. The plot in Fig. 5a shows *Q* as a function of *p*_1_ and *p*_2_ for two distinct binding scenarios. First, if the *K*_d_ value of 2 mM obtained in this work is the total, macroscopic dissociation constant for the low-affinity complex (*K*_d,tot_), comprising both the dominant and minor species, then *p*_1_+*p*_2_=1. In this case, the overall amount of CL bound to Cc is independent of the relative populations of the two binding forms. In contrast, if the measured *K*_d_ value is the microscopic dissociation constant for the dominant binding site only (*K*_d,1_), then the fraction of bound CL rises with increasing *p*_2_ (that is, *p*_1_+*p*_2_>1 at fixed *p*_1_=1). The results of molecular refinement corresponding to the two situations are presented in the left- and right-hand side plots of the Fig. 5a, respectively. The *Q* values in the right panel are consistently smaller than those on the left, with the minimal values located in the region of *p*_2_=0.6−0.9 at the constant *p*_1_=1.0 indicating the best agreement with the experimental data. Thus, comparative analysis of the *Q* factors allows to discriminate between the two binding scenarios. Moreover, the determined populations can be converted into the microscopic dissociation constants for the two binding forms, *K*_d,1_=2.0±0.4 mM and *K*_d,2_=2.7±0.5 mM, as well as the overall, macroscopic *K*_d,tot_=1.2±0.2 mM for the low-affinity complex (Supplementary Note 1 and Supplemetary Fig. 7). As demonstrated in earlier studies^35^,^36^ and mentioned above, chemical shift perturbations are largely insensitive to the presence of minor species comprising multiple, transient protein–protein orientations and report mainly on the dominant form of the complex. This explains why the Δ*δ* titrations performed here provided the microscopic dissociation constant for the dominant CL–Cc form only, as confirmed by the PRE-based *Q*-factor analysis.

The ensemble refinement of the minor Cc–CL form consistently produced solutions with bound Cc molecules populating two spatial regions: one bordering CcP residues D148 and D217 and extending to the surface patch containing D33 and E35, and the other defined by the CcP residues E167, D261 and E267 (Fig. 5b). Multiple Cc conformers of the minor species do not overlap with the Cc molecule in the dominant Cc–CL form, even though such overlap was allowed in the ensemble refinement protocol. This finding validates the present quantitative analysis of the low-affinity Cc–CL complex with the binding model for two individual, non-overlapping sites. Extending these conclusions to the native, non-covalent Cc–CcP complex, and borrowing the terminology of Hoffman and co-workers^11^, we can say that CcP harbours two domains—those binding Cc with high and low affinity and yielding, respectively, the crystallographic Cc–CcP form and the weak complex studied here. The low-affinity domain contains two binding sites comprising the dominant protein–protein orientation and an ensemble of lowly populated binding geometries, which summarily account for the observed PRE effects. Such dynamic view of the ternary CcP–(Cc)_2_ complex agrees with conclusions of earlier computational studies and very recent ET kinetics work^32^,^37^.

The equilibrium populations of different bound species, determined from microscopic *K*_d_s for the two low-affinity sites obtained in this work and the reported *K*_d_ values for the high-affinity domain^1^, can be converted into the corresponding binding energies, schematically depicted in the energy diagram (inset in Fig. 5a). Previous experimental studies suggested that electrostatic repulsion between two Cc molecules in the ternary complex accounts for the drastic difference in the affinity constants for the first and second binding steps^37^,^38^,^39^. Difference in binding energies for the high- and low-affinity sites (ΔΔ*G*) provides the upper limit for the electrostatic repulsion energy (Δ*G*_Φ_), a presumed dominant term in the energy penalty for such ‘anticooperative' binding. The value of ΔΔ*G*≤5.5 kcal mol^−1^ calculated in this work can be compared with those of Δ*G*_Φ_=6 kcal mol^−1^ and Δ*G*_Φ_≤3.3 kcal mol^−1^ obtained from steady-state kinetics and flash photolysis experiments conducted under similar experimental conditions^37^,^39^.

### Experimental validation of the observed binding effects

**Figure 6:**
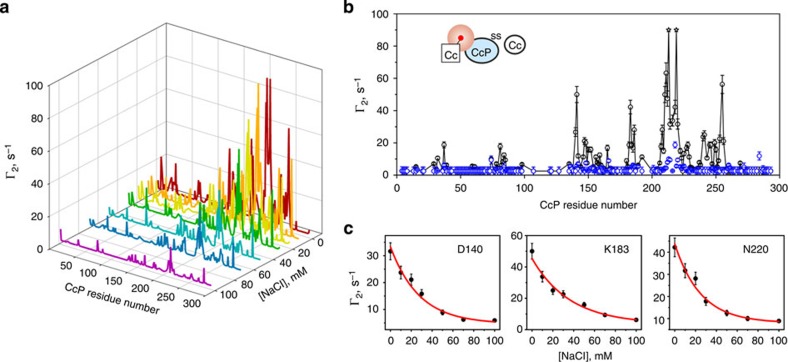
Effect of the ionic strength on the intermolecular PREs in the CL–Cc system. (**a**) CcP-observed Γ_2_ PREs for the CL in the presence of 3 equivalents of Cc E88C-EDTA(Mn^2+^) in 20 mM NaP_i_ pH 6.0 at varying concentrations of NaCl. (**b**) Comparison of the PRE profiles obtained at 0 mM (black) and 100 mM (blue) NaCl. Stars indicate the residues whose resonances disappear in the paramagnetic spectrum. The errors are s.d. The inset schematically depicts the experimental set-up, with the NMR-active protein coloured light blue, and the attached paramagnetic label indicated by the red sphere. (**c**) Ionic strength dependence of Γ_2_ for several CL CcP residues. The errors are s.d. The red lines show the best fits to an exponential decay function with the decay rates of 28±4 (D140, *r*^2^=0.97), 36±6 (K183, *r*^2^=0.98) and 25±4 (N220, *r*^2^=0.98).

**Figure 7:**
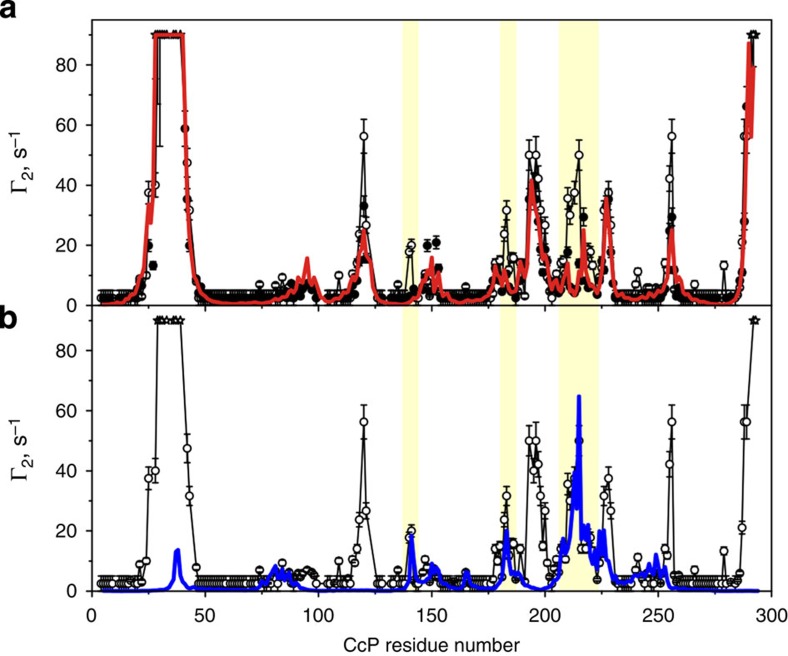
PREs for the native non-covalent Cc–CcP complexes at low and high ionic strengths. (**a**,**b**) Intermolecular PREs for the backbone amide resonances of [D, ^15^N] wt CcP interacting with Cc E88C-EDTA(Mn^2+^) in 20 mM NaP_i_ pH 6.0 and [NaCl]=0 mM (open symbols) or [NaCl]=100 mM (filled symbols). The high and low-salt samples contained 0.4 mM CcP and 1 or 3 equivalents of Cc, respectively. The high-salt data were taken from our previous work^28^. The red line in **a** shows the Γ_2_ values calculated for the combination of the high-affinity binding orientation and the encounter complex at [NaCl]=100 mM^28^. The blue line in (**b**) represents the PREs calculated for the combination of the dominant binding geometry and multiple, lowly populated protein–protein orientations constituting the low-affinity Cc–CcP complex (studied in this work) and corresponds to the red trace in Fig. 3a. Several regions exhibiting differences between the high- and low-salt PRE profiles are highlighted.

Analysis of the intermolecular PREs at different ionic strengths confirms that the Cc binding to the low-affinity CcP domain is salt sensitive (Fig. 6). For most of the CL CcP residues, the paramagnetic effect decreases exponentially with the increasing salt concentration, yielding essentially flat Γ_2_ profiles at 100 mM NaCl (Fig. 6a, b). Thus, in agreement with earlier studies^1^,^2^,^12^, the low-affinity binding is abolished at 100 mM salt. Consistent with this finding, the effects originating from the low-affinity domain account for most of the differences between the PRE profiles of the native, non-covalent Cc–CcP complex at high and low ionic strengths (Fig. 7). At 100 mM NaCl, the measured PREs are well represented by a combination of effects from the high-affinity binding geometry and its transient encounter state (Fig. 7a)^28^. Reflecting the higher fraction of CcP bound, the corresponding Γ_2_ values at 0 mM NaCl are consistently larger, but for the most part follow the same pattern as those at high ionic strength, suggesting that the structure of the Cc–CcP complex and its encounter state remains the same (Fig. 7b). Manifested as additional contributions to the low-salt PRE data, the main differences between the two Γ_2_ profiles are found for the CcP regions that sense Cc binding to the low-affinity domain (highlighted in Fig. 7). These results show that the observed effects are not specific to the Cc–CL system, but are also found in the native, non-covalent complex.

**Figure 8:**
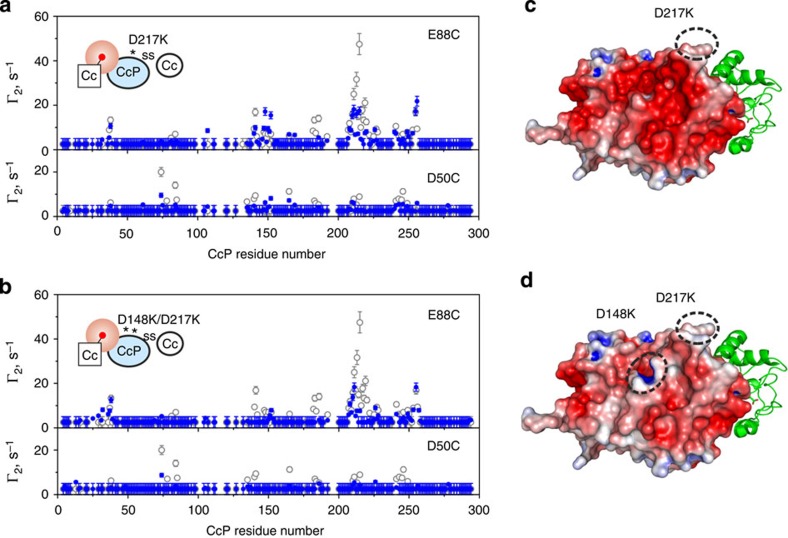
PRE NMR analysis of the Cc interaction with the charge-reversal CcP–Cc CLs. (**a**,**b**) Intermolecular, CcP-observed PREs caused by the binding of the paramagnetically labelled E88C and D50C Cc to (**a**) D217K/V197C and (**b**) D148K/D217K/V197C [D, ^13^C, ^15^N] CcP–A81C Cc CLs. The measured PREs (blue symbols) are compared with those of the original, ‘wt' CL (open symbols; also shown in Fig. 3a). The errors are s.d. The insets schematically depict the experimental set-up, with the NMR-active protein coloured light blue, the attached paramagnetic label indicated by the red sphere and point mutations represented by asterisks. (**c**,**d**) The charge-reversal CLs with the molecular surface of CcP coloured by the electrostatic potential (from −5 *k*_B_*T* in red to +5 *k*_B_*T* in blue, calculated with APBS)^57^. The protein orientations are the same as in Fig. 2h. The introduced mutations are indicated by dashed circles. All NMR samples contained 0.5 mM CL and 1 equivalents of Cc in 20 mM NaP_i_ pH 6.0.

Finally, to validate the location of the low-affinity binding site and assess the role of CcP residues D148 and D217 in the CL–Cc complex formation, we prepared two charge-reversal CL variants, in which either one or both of the aspartates are substituted by lysines. PRE NMR experiments with D217K and D148K/D217K CLs show that Cc-binding effects are greatly reduced in the former and even more so in the latter system (Fig. 8). A large decrease is observed for the intermolecular PREs originating from both the dominant and minor CL–Cc forms, which can be attributed to changes in the electrostatic properties of CcP in the variant CLs. These results illustrate the importance of D148 and D217 residues for the low-affinity Cc–CcP complex formation and confirm that the negatively charged region of the CcP surface identified in the classic Brownian dynamics study^32^ is indeed the location of the low-affinity binding site.

### ET properties of the low-affinity site

**Figure 9:**
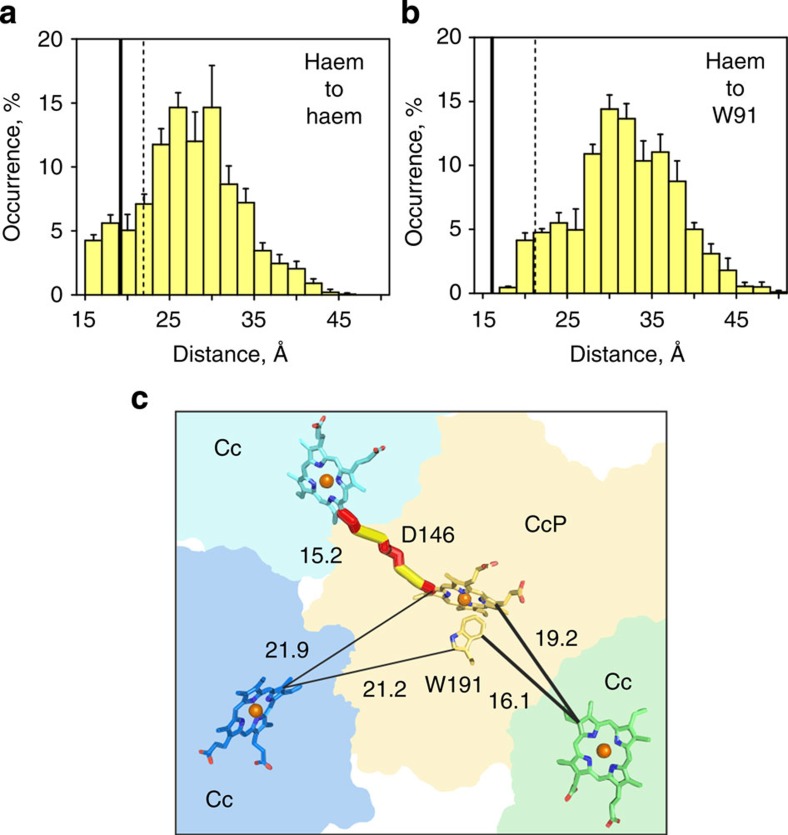
ET properties of the low-affinity Cc–CcP complex. (**a**,**b**) Distributions of the edge-to-edge (**a**) haem–haem and (**b**) Cc haem–CcP W191 distances in the low-affinity complex. The values are averaged over the data sets highlighted in Fig. 5a; the errors are s.d. The solid and dashed lines indicate the corresponding distances in the crystallographic orientation and the dominant low-affinity binding form, respectively. (**c**) Shortest edge-to-edge separations (in Å) among the redox centres in the high-affinity (thick lines) and low-affinity (thin lines and solid cylinders) complexes. Surface outlines of CcP and Cc in the crystallographic orientation are coloured yellow and green, while those of the dominant form and a representative ET active geometry of the low-affinity complex are in blue (bottom left) and cyan (top left), respectively. In the latter, the solid cylinders indicate the intermolecular ET pathway mediated by the covalent bonds (red) of the haem groups and the intervening CcP residue D146 and two through-space jumps (yellow).

In a broad range of biological systems, the ET rate constants are described by an exponential dependence on the distances between the redox centres^40^. Thus, to assess the ET properties of the low-affinity Cc–CcP complex, we analysed the separations between the haem group of Cc and two redox centres in CcP CpdI—the haem oxyferryl and W191 cation-radical (Fig. 9). Judging from the large distances of 21 Å (haem–W191) and 22 Å (haem–haem), the dominant low-affinity binding geometry is inactive in the intermolecular ET. In contrast, the conformational ensemble constituting the minor form contains multiple, ET-competent protein–protein orientations with short haem–haem separations of <16 Å (Fig. 9a). Calculated from the edge-to-edge haem-to-haem distances in the four simulated ensembles (highlighted in Fig. 5a) as the population-weighted average over all Cc–CcP orientations constituting the low-affinity complex, the ET rate (*k*_ET_) is found to be 1,324–2,343 s^−1^. The overall average value of <*k*_ET_>=1,950±450 s^−1^ can be compared with the experimentally measured *k*_ET_=1,540±80 s^−1^ for direct haem–haem ET from the low-affinity domain in the 2:1 Cc–CcP complex^10^,^11^. As the high-affinity crystallographic orientation and the encounter state exhibit large separations between the prosthetic groups^7^,^28^, it appears that the low-affinity domain alone accounts for the measured haem–haem ET activity^8^,^9^,^10^,^11^.

The low-affinity complex studied here is adequately described by the ‘dynamic docking' model of protein–protein interactions^41^, in which numerous, lowly populated Cc–CcP orientations contribute to the overall binding, but only a small number of these are ET active. Moreover, as illustrated by computational studies, which delineated the ET-competent regions of the Cc–CcP conformational space^42^ and mapped out the ET coupling pathways to the protein surfaces^11^, the most ET active orientation is not necessarily the most thermodynamically stable. An example of a transient, ET-competent Cc–CcP orientation is given in Fig. 9c. With the haem-to-haem separation of 15 Å, this bound form is expected to be highly ET active. Indeed, analysis of the electronic tunnelling coupling with the model developed by Beratan *et al.*^43^ identified the optimal ET pathway with the overall *k*_ET_ rate of 3.6 × 10^5^ s^−1^. Originating at the conjugated π-system of the Cc haem, the pathway proceeds via two covalent bonds of the thioether-bound 4-ethylene substituent, followed by a through-space jump to the Oδ_2_ atom of the CcP residue D146 and a travel along its Oδ_2_-C_γ_-C_β_-C_α_-C^′^ covalent bonds, followed by another through-space jump to the CcP haem 1-methyl (Fig. 9c).

Taken at the face value, the existence of the ET-competent binding geometries seems to contradict the findings of Erman and co-workers, who showed that covalent Cc–CcP complexes with the blocked high-affinity site—CcP(V197C)-Cc(A81C), primarily studied here, and CcP(E290C)-Cc(K73C), the first site-specific CL designed to probe the ET activity at the low-affinity site^24^—are inactive towards externally added Cc^29^. However, the fact that no enzyme turnover is observed while the haem-to-haem ET is feasible confirms that the catalytic cycle involves ET to the W191 cation-radical^1^,^2^,^29^ and highlights the central role of this CpdI redox intermediate in the CcP function. In agreement with this conclusion, the low-affinity complex displays large separations between Cc haem and CcP W191 groups (Fig. 9b), indicating that it does not support the functionally relevant ET activity. This finding is also consistent with the studies of Hoffman and co-workers, as the ET observed in their flash photolysis experiments occurs directly between two haem groups, bypassing formation of W191^+·^ species^8^,^9^,^10^,^11^. While being of great academic interest, the low-affinity Cc–CcP binding appears to be irrelevant for the physiological function. As this interaction is abolished under the physiological, high ionic strength conditions, the cellular enzymatic activity of CcP relies solely on the intermolecular ET to CpdI W191^+·^, taking place from the high-affinity, crystallographic Cc–CcP orientation.

## Methods

### Protein samples

Single-cysteine mutants of yeast Cc and CcP were prepared by site-directed mutagenesis using whole-plasmid synthesis PCR (WHOPS PCR) protocol^44^, starting from the DNA sequences coding for proteins with substituted native cysteine residues (T-5A/C102T Cc, referred to as the wt Cc^45^, and C128A CcP, prepared in a separate WHOPS PCR step). Charge-reversal CcP mutants D217K/V197C/C128A and D148K/D217K/V197C/C128A were prepared by additional WHOPS PCRs on the V197C/C128A CcP DNA template. All constructs were verified by DNA sequencing. Expression vectors for the wt proteins, Cc variants D50C, E66C and E88C, and the single-cysteine D32C Ub were prepared before^25^,^28^,^45^.

The natural-abundance wt proteins and their single-cysteine variants, uniformly labelled [D, ^15^N] wt and V197C/C128A CcP and ^15^N-labelled wt Cc were produced in *Escherichia coli* and purified following published procedures^25^,^28^,^45^. The EDTA(Mn^2+^) paramagnetic probe was attached via cysteine reaction with Mn^2+^ complex of N-[S-(2-pyridylthio)cysteaminyl] ethylenediamine-*N*,*N*,*N*′,*N*′-tetraacetate monoamide (Toronto Research Chemicals), and the conjugated products were purified and characterized as described before^28^.

The protein concentrations were calculated from the ultraviolet–visible spectra using the known extinction coefficients for the Soret band absorption maxima^14^,^25^,^45^. Complexes of the low-spin CcP(CN) and Cc (Fe^2+^), mimicking the CpdI^46^, were studied throughout this work. All NMR samples were prepared in 20 mM NaP_i_ pH 6.0 and contained 6 % D_2_O for the lock. Unless stated otherwise, the PRE measurements were conducted on 0.4 mM [D, ^15^N] CcP–Cc CL and 1 equivalents of the Cc-EDTA(Mn^2+^) (for the paramagnetic samples) or wt Cc (for the diamagnetic reference). The compositions of the samples used for the control experiments (Figs 4 and 6, 7, 8 and Supplementary Fig. 5) are indicated in the corresponding figure legends.

### Synthesis of the disulfide CLs

To obtain V197C CcP–A81C Cc and E290C CcP–K73C Cc CLs in high yield, we adopted the strategy of Otting and co-workers^23^, schematically shown in Supplementary Fig. 6. First, to reduce the possible intermolecular disulfides, a relevant single-cysteine CcP variant was incubated with a 10-fold molar excess of DTT for 1 h at room temperature (RT) and then exchanged into 20 mM Tris-HCl 100 mM NaCl pH 8.0 on a HiTrap Desalting column (GE Healthcare), concomitantly removing the reductant. Second, the CcP—now bearing a free thiol group—was incubated with a 10-fold molar excess of 5,5′-dithiobis-(2-nitrobenzoate) [DTNB or Ellman's reagent] for 1 h at RT, yielding the modified protein and the yellow-coloured TNB. The unreacted DTNB and the TNB product were removed on a desalting column, leaving the protein solution containing the CcP-TNB adduct. Third, a fivefold molar excess (relative to CcP) of the corresponding single-cysteine Cc variant was incubated with a 10-fold molar excess (relative to Cc) of DTT for 1 h at RT, exchanged into 20 mM Tris-HCl 100 mM NaCl pH 8.0 on a desalting column and combined with the CcP-TNB solution. The cross-linking reaction between the free thiol-bearing Cc and the TNB-activated CcP was carried out for 16–18 h at RT, yielding the desired disulfide-linked CcP–Cc heterodimer (Supplementary Fig. 6). Finally, the protein CL was purified from the reaction mixture by ion-exchange chromatography and characterized by SDS–PAGE, ultraviolet–visible spectrophotometry and analytical size-exclusion chromatography (Fig. 1 and Supplementary Figs 1 and 2). The analytical gel-filtration experiments were performed on an ENrich SEC 70 column (Bio-Rad) equilibrated in 20 mM sodium phosphate (NaP_i_) 100 mM NaCl pH 6.0 and run at a flow rate of 0.5 ml min^−1^. The CL samples were injected at a concentration of 0.25 mM. The Bio-Rad size-exclusion standard (a lyophilized mixture of molecular weight markers ranging from 1.35 to 670 kDa) was used to calibrate the column. Two charge-reversal CL variants, D217K/V197C and D148K/D217K/V197C CcP–A81C Cc containing the uniformly labelled [D, ^13^C, ^15^N] CcP, were prepared by the same procedure, yielding the desired pure products (Supplementary Fig. 2).

### NMR experiments and data analysis

The NMR experiments were conducted at 298 K on a Varian NMR Direct-Drive System 600 MHz spectrometer (^15^N Cc-observed CL titrations) or an 800-MHz spectrometer equipped with either a RT or a salt-tolerant PFG-Z cold probe (all other experiments). The NMR data were processed in NMRPipe^47^ and analysed in CCPN^48^. The assignments of the backbone amide resonances of [D, ^15^N] CcP(CN) and ^15^N Cc were taken from our earlier work^25^,^28^,^45^. For most of the CcP resonances in [D, ^15^N] CcP–Cc CLs, the assignments could be transferred directly from the spectra of the free protein (see Results section above). The assignments of the charge-reversal CL variants, D217K/V197C and D148K/D217K/V197C CcP–A81C Cc containing the uniformly labelled [D, ^13^C, ^15^N] CcP, were verified by TROSY-selected, deuterium-decoupled 3D HNCA, HN(CO)CA, and out-and-back HN(CA)CB experiments. Except for several resonances of residues in and around the mutation sites, the 2D [^1^H, ^15^N] TROSY spectra of the variant CLs are virtually identical to that of the original [D, ^15^N] V197C CcP–A81C Cc construct, indicating that the introduced point mutations do not perturb the CcP structure.

NMR titrations were performed by incremental addition of the concentrated Cc stock solution (2.6–2.9 mM) to the CcP–Cc CL samples at the initial concentrations of 0.32–0.46 mM. At each increment, chemical shift perturbations of backbone amide resonances of either [D, ^15^N] CcP–Cc CL or ^15^N Cc were monitored in 2D [^1^H, ^15^N] correlation spectra. The titration curves were analysed with a two-parameter non-linear least-squares fit using a one-site binding model corrected for the dilution effect^46^. The average chemical shift perturbations were calculated as Δ*δ*=(Δ*δ*_N_^2^/50+Δ*δ*_H_^2^/2)^0.5^, where Δ*δ*_N_ and Δ*δ*_H_ are the chemical shift perturbations of the backbone amide nitrogen and proton, respectively, for a given protein residue.

The ^1^H Γ_2_ PREs were obtained from two identical [^1^H, ^15^N] TROSY-selected heteronuclear single-quantum correlation experiments with the relaxation delay of 10 s run on the paramagnetic and diamagnetic samples. The resonances showing strong spectral overlap were excluded from the analysis. The ratios of the signal intensities were converted into the Γ_2_ values, and the Γ_2_ errors were propagated from the signal intensities and the spectral noise levels as described in our recent report^28^.

### Ensemble refinement against intermolecular PREs

The coordinates of the Cc–CcP CL were taken from the X-ray structure (PDB ID 1S6V)^14^. Using the PRE data set obtained from the three EDTA(Mn^2+^) conjugation sites (D50C, E66C and E88C Cc), the rigid-body simulated annealing refinement of the Cc–CL complex was carried out in Xplor-NIH^49^,^50^ following the published procedure^34^. In brief, the position of the CL was fixed, and either a single or multiple copies of the Cc molecule were docked to minimize the energy function consisting of the PRE target term, van der Waals repulsion term to prevent atomic overlap between Cc and CL, and a weak radius-of-gyration restraint used to encourage intermolecular Cc–CL contacts^34^. Note that this procedure allows for the atomic overlap among Cc molecules constituting an ensemble, as well as among the Cc ensemble members and the Cc of the dominant bound form. To account for the mobility of the attached label, the calculated effects were averaged over an ensemble of 50 EDTA(Mn^2+^) conformers generated by simulated annealing in torsion angle space^51^. As explained in the text, the relative populations of the two bound species, *p*_1_ and *p*_2_, were varied in multiple refinement runs. As a rule, 100 independent calculations were performed in each run, and 50 solutions with lowest *Q* factors (see below) were selected for further analysis.

To assess the agreement between the observed PREs and the PREs back-calculated from Cc ensembles generated in each run, we calculated the *Q* factor^34^ (equation 1):


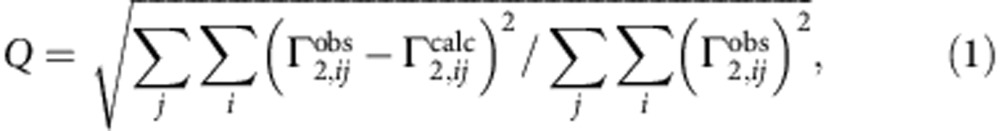


where *j*=1–3 runs over the three EDTA(Mn^2+^) attachment sites on Cc and 
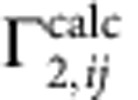
 is given by equation 2:


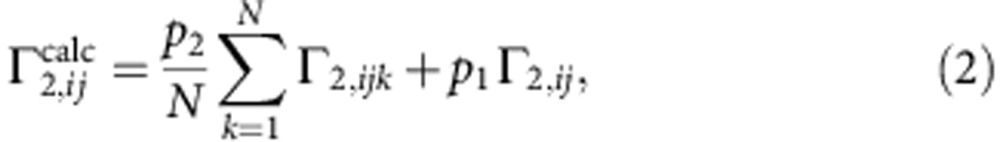


where *p*_1_ and *p*_2_ are the total populations of the dominant and minor Cc–CL species, respectively, *N* is the ensemble size of the latter, Γ_2,*ijk*_ is the PRE from EDTA(Mn^2+^) (*j*) back-calculated for the residue (*i*) of the Cc ensemble member (*k*), and Γ_2,*ij*_ is the PRE back-calculated from EDTA(Mn^2+^) (*j*) for the residue (*i*) of Cc in the dominant form of the complex.

### ET rate calculations

The rate constants for the intermolecular ET, *k*_ET_, were calculated from the edge-to-edge distances between the haem groups (equation 3)^40^:


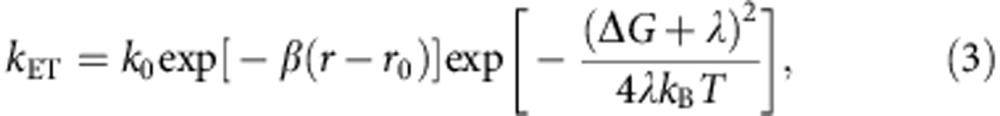


where *k*_0_=10^13^ s^−1^ is the nuclear frequency, 
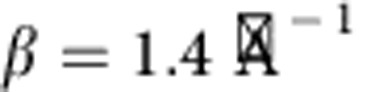
 is the decay coefficient of the electronic coupling, *r* is the edge-to-edge distance between the redox centres, *r*_0_=3.6 Å is the van der Waals contact distance, Δ*G* is the free energy difference between reactant and product states, *λ* is the reorganization energy, *k*_B_ is the Boltzmann constant and *T* is the temperature. The values of Δ*G*=−0.797 eV and *λ*=0.7 eV were taken from the literature^42^,^52^. As shown by Marcus and Sutin^53^ and further confirmed by density functional theory calculations of the electron density distribution in the haem derivatives^54^,^55^, mixing of the t_2g_-orbitals of the Fe center in Cc with the π*-orbitals of the porphyrin ring effectively extends the metal *d*-electron density to the porphyrin edge, which justifies the use of edge-to-edge distances in the ET analysis of the Cc–CcP system. The overall average <*k*_ET_> reported in the text was obtained from the *k*_ET_ rates of the Cc–CcP complexes generated in four ensemble refinement runs with the smallest *Q* factors (highlighted in Fig. 5a), calculated as population-weighted averages of the *k*_ET_ values for individual protein–protein orientations in the 50 best Cc–CcP solutions.

Alternatively, the *k*_ET_ was derived from the analysis of the ET pathways (equation 4)^56^:


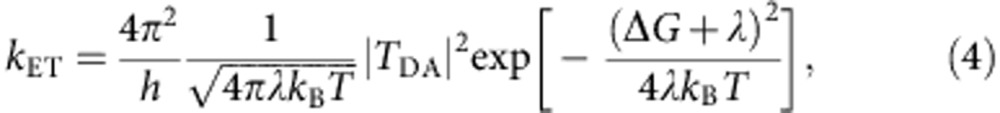


where *h* is the Planck constant, *T*_DA_ is the electronic donor-to-acceptor tunnelling coupling and the other symbols are the same as above. The *T*_DA_ factor can be estimated using the Pathways model^43^, which treats electron tunnelling as a sequence of steps taking place through a covalent bond, a hydrogen bond, or the vacuum, and represents the *T*_DA_ as the product of penalties for each step, equation 5 (ref. 56):


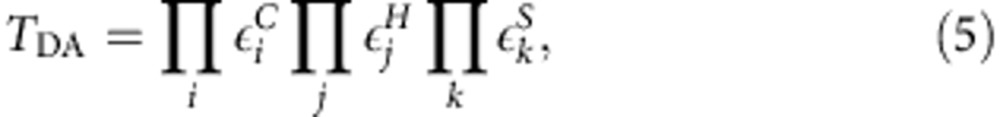


where *ɛ*^C^=0.6 is the penalty for the covalent bond-mediated step; 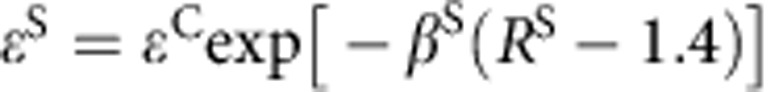 is the penalty for a through-space jump, where *R*^S^ is the jump distance in Å; 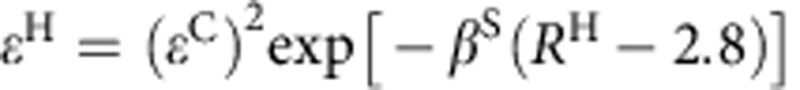 is the penalty for a hydrogen bond-mediated step, where *R*^H^ is the hydrogen bond length in Å; and 
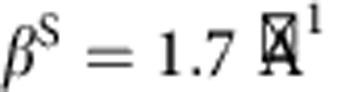
 is the decay factor for the vacuum^56^. The search for the ET pathways and the *T*_DA_ calculations were carried out with the Pathways plugin for the molecular visualization programme VMD^56^. In general, the *k*_ET_ values calculated from the ET distances (equation 3) or obtained with the Pathways model (equations 4 and 5) in this work agreed to within a factor of two.

## Author contributions

A.N.V. conceived and designed the experiments; K.V.d.W. and A.N.V. performed the experiments; K.V.d.W., Y.G.J.S. and A.N.V. analysed the data; A.N.V. wrote the paper with contributions from all authors.

## Additional information

**Accession Codes:** An NMR ensemble of 15 lowest-energy CL-Cc structures has been deposited in the Protein Data Bank under the code 2N18.

**How to cite this article:** Van de Water, K. *et al.* The low-affinity complex of cytochrome *c* and its peroxidase. *Nat. Commun.* 6:7073 doi: 10.1038/ncomms8073 (2015).

## Supplementary Material

Supplementary Information Supplementary Figures 1-7, Supplementary Table 1, Supplementary Note 1 and Supplementary References

## References

[b1] Erman J. E. & Vitello L. B. Yeast cytochrome *c* peroxidase: mechanistic studies via protein engineering. Biochim. Biophys. Acta 1597, 193–220 (2002). 12044899 10.1016/s0167-4838(02)00317-5

[b2] Volkov A. N., Nicholls P. & Worrall J. A. R. The complex of cytochrome *c* and cytochrome *c* peroxidase: the end of the road? Biochim. Biophys. Acta 1807, 1482–1503 (2011). 21820401 10.1016/j.bbabio.2011.07.010

[b3] Miller M.A. *et al.* Identifying the physiological electron transfer site of cytochrome *c* peroxidase by structure-based engineering. Biochemistry 35, 667–673 (1996). 8547245 10.1021/bi952557a

[b4] Wang K. *et al.* Design of a ruthenium-cytochrome *c* derivative to measure electron transfer to the radical cation and oxyferryl heme in cytochrome *c* peroxidase. Biochemistry 35, 15107–15119 (1996). 8942678 10.1021/bi9611117

[b5] Miller M. A. A complete mechanism for steady-state oxidation of yeast cytochrome *c* by yeast cytochrome *c* peroxidase. Biochemistry 35, 15791–15799 (1996). 8961942 10.1021/bi961488c

[b6] Mei H. *et al.* Control of formation and dissociation of the high-affinity complex between cytochrome *c* and cytochrome *c* peroxidase by ionic strength and the low-affinity binding site. Biochemistry 35, 15800–15806 (1996). 8961943 10.1021/bi961487k

[b7] Pelletier H. & Kraut J. Crystal structure of a complex between electron transfer partners, cytochrome *c* peroxidase and cytochrome *c*. Science 258, 1748–1755 (1992). 1334573 10.1126/science.1334573

[b8] Stemp E. D. A. & Hoffman B. M. Cytochrome *c* peroxidase binds two molecules of cytochrome *c*: evidence for a low-affinity, electron-transfer-active site on cytochrome *c* peroxidase. Biochemistry 32, 10848–10865 (1993). 8399235 10.1021/bi00091a041

[b9] Zhou J. S. & Hoffman B. M. Cytochrome *c* peroxidase simultaneously binds cytochrome *c* at two different sites with strikingly different reactivities: titrating a "substrate" with an enzyme. J. Am. Chem. Soc. 115, 11008–11009 (1993).

[b10] Zhou J. S. & Hoffman B. M. Stern-Volmer in reverse: 2:1 stoichiometry of the cytochrome *c* - cytochrome *c* peroxidase electron-transfer complex. Science 265, 1693–1696 (1994). 8085152 10.1126/science.8085152

[b11] Nocek J. M. *et al.* Theory and practice of electron transfer within protein-protein complexes: application to multidomain binding of cytochrome *c* by cytochrome *c* peroxidase. Chem. Rev. 96, 2459–2490 (1996). 11848833 10.1021/cr9500444

[b12] Mauk M. R., Ferrer J. C. & Mauk A. G. Proton linkage in formation of the cytochrome *c* - cytochrome *c* peroxidase complex: electrostatic properties of the high- and low-affinity cytochrome binding sites on the peroxidase. Biochemistry 33, 12609–12614 (1994). 7918486 10.1021/bi00208a011

[b13] Morar A. S. & Pielak G. J. Crowding by trisaccharides and the 2:1 cytochrome *c* - cytochrome *c* peroxidase complex. Biochemistry 41, 547–551 (2002). 11781093 10.1021/bi0111810

[b14] Guo M., Bhaskar B., Li H., Barrows T. P. & Poulos T. L. Crystal structure and characterization of a cytochrome *c* peroxidase - cytochrome *c* site-specific cross-link. Proc. Natl Acad. Sci. USA 101, 5940–5945 (2004). 15071191 10.1073/pnas.0306708101 PMC395902

[b15] Tang C., Ghirlando R. & Clore G. M. Visualization of transient ultra-weak protein self-association in solution using paramagnetic relaxation enhancement. J. Am. Chem. Soc. 130, 4048–4056 (2008). 18314985 10.1021/ja710493m

[b16] Johansson H. *et al.* Specific and nonspecific interactions in ultraweak protein-protein associations revealed by solvent paramagnetic relaxation enhancements. J. Am. Chem. Soc. 136, 10277–10286 (2014). 24969589 10.1021/ja503546j PMC4111215

[b17] Liu Z. *et al.* Noncovalent dimerization of ubiquitin. Angew. Chem. Int. Ed. 51, 469–472 (2012). 10.1002/anie.201106190 PMC3303887 22109817

[b18] Xing Q. *et al.* Visualizing an ultra-weak protein-protein interaction in phosphorylation signaling. Angew. Chem. Int. Ed. 53, 11501–11505 (2014). 10.1002/anie.201405976 25131700

[b19] Gustavsson M. *et al.* Allosteric regulation of SERCA by phosphorylation-mediated conformation shift of phospholamban. Proc. Natl Acad. Sci. USA 110, 17338–17343 (2013). 24101520 10.1073/pnas.1303006110 PMC3808617

[b20] Fusco G. *et al.* Direct observation of the three regions in α-synuclein that determine its membrane-bound behaviour. Nat. Commun. 5, 3827 (2014). 24871041 10.1038/ncomms4827 PMC4046108

[b21] Clore G. M. Visualizing lowly-populated regions of the free energy landscape of macromolecular complexes by paramagnetic relaxation enhancement. Mol. Biosyst. 4, 1058–1069 (2008). 18931781 10.1039/b810232e PMC2807640

[b22] Anthis N. J. & Clore G. M. Visualizing transient dark states by NMR spectroscopy. Q. Rev. Biophys. 48, 35–116 (2015). 25710841 10.1017/S0033583514000122 PMC6276111

[b23] Su X. C., Huber T., Dixon N. E. & Otting G. Site-specific labelling of proteins with a rigid lanthanide-binding tag. Chem. Bio. Chem. 7, 1599–1604 (2006). 10.1002/cbic.200600142 16927254

[b24] Pappa H. S. & Poulos T. L. Site-specific cross-linking as a method for studying intramolecular electron transfer. Biochemistry 34, 6573–6580 (1995). 7756288 10.1021/bi00020a001

[b25] Volkov A. N., Wohlkonig A., Soror S. H. & van Nuland N. A. J. Expression, purification, characterization, and solution nuclear magnetic resonance study of highly-deuterated yeast cytochrome *c* peroxidase with enhanced solubility. Biochemistry 52, 2165–2175 (2013). 23517193 10.1021/bi400220w

[b26] Volkov A. N., Worrall J. A. R., Holtzmann E. & Ubbink M. Solution structure and dynamics of the complex between cytochrome *c* and cytochrome *c* peroxidase determined by paramagnetic NMR. Proc. Natl Acad. Sci. USA 103, 18945–18950 (2006). 17146057 10.1073/pnas.0603551103 PMC1748157

[b27] Volkov A. N., Ubbink M. & van Nuland N. A. J. Mapping the encounter state of a transient protein complex by PRE NMR spectroscopy. J. Biomol. NMR 48, 225–236 (2010). 21049303 10.1007/s10858-010-9452-6 PMC3235994

[b28] Van de Water K., van Nuland N. A. J. & Volkov A. N. Transient protein encounters characterized by paramagnetic NMR. Chem. Sci. 5, 4227–4236 (2014).

[b29] Nakani S., Viriyakul T., Mitchell R., Vitello L. B. & Erman J. E. Characterization of a covalently linked yeast cytochrome *c* - cytochrome *c* peroxidase complex: evidence for a single, catalytically active cytochrome *c* binding site on cytochrome *c* peroxidase. Biochemistry 45, 9887–9893 (2006). 16893189 10.1021/bi060586n

[b30] Riddles P. W., Blakeley R. L. & Zerner B. Ellman's reagent: 5,5'-dithiobis(2-nitrobenzoic acid) - a reexamination. Anal. Biochem. 94, 75–81 (1979). 37780 10.1016/0003-2697(79)90792-9

[b31] Hall D. A. *et al.* Mapping the interactions between flavodoxin and its physiological partners flavodoxin reductase and cobalamin-dependent methionine synthase. Proc. Natl Acad. Sci. USA 98, 9521–9526 (2001). 11493691 10.1073/pnas.171168898 PMC55485

[b32] Northrup S. H., Boles J. O. & Reynolds J. C. L. Brownian dynamics of cytochrome *c* and cytochrome *c* peroxidase association. Science 241, 67–70 (1988). 2838904 10.1126/science.2838904

[b33] Vinogradova O. & Qin J. NMR as a unique tool in assessment and complex determination of weak protein-protein interactions. Top. Curr. Chem. 326, 35–45 (2012). 21809187 10.1007/128_2011_216 PMC3676910

[b34] Tang C., Iwahara J. & Clore G. M. Visualization of transient encounter complexes in protein-protein association. Nature 444, 383–386 (2006). 17051159 10.1038/nature05201

[b35] Worrall J. A. R., Reinle W., Bernhardt R. & Ubbink M. Transient protein interactions studied by NMR spectroscopy: the case of cytochrome *c* and adrenodoxin. Biochemistry 42, 7068–7076 (2003). 12795602 10.1021/bi0342968

[b36] Xu X. *et al.* Dynamics in a pure encounter complex of two proteins studied by solution scattering and paramagnetic NMR spectroscopy. J. Am. Chem. Soc. 130, 6395–6403 (2008). 18439013 10.1021/ja7101357

[b37] Page T. R. & Hoffman B. M. Control of cyclic photoinitiated electron transfer between cytochrome *c* peroxidase (W191F) and cytochrome *c* by formation of dynamic binary and ternary complexes. Biochemistry 54, 1188–1197 (2015). 25629200 10.1021/bi500888y PMC4413949

[b38] McLendon G. *et al.* Thermodynamic and kinetic aspects of binding and recognition in the cytochrome *c* / cytochrome *c* peroxidase complex. J. Am. Chem. Soc. 115, 3665–3669 (1993).

[b39] Nakani S., Vitello L. B. & Erman J. E. Characterization of four covalently-linked yeast cytochrome *c* / cytochrome *c* peroxidase complexes: evidence for electrostatic interaction between bound cytochrome *c* molecules. Biochemistry 45, 14371–14378 (2006). 17128976 10.1021/bi061662p PMC2556041

[b40] Moser C. C., Keske J. M., Warncke K., Farid R. S. & Dutton P. L. Nature of biological electron transfer. Nature 355, 796–802 (1992). 1311417 10.1038/355796a0

[b41] Liang Z. X. *et al.* Dynamic docking and electron transfer between Zn-myoglobin and cytochrome *b*_5_. J. Am. Chem. Soc. 124, 6849–6859 (2002). 12059205 10.1021/ja0127032

[b42] Volkov A. N. & van Nuland N. A. J. Electron transfer interactome of cytochrome *c*. PLoS Comput. Biol. 8, e1002807 (2012). 23236271 10.1371/journal.pcbi.1002807 PMC3516563

[b43] Beratan D. N., Betts J. N. & Onuchic J. N. Protein electron transfer rates set by the bridging secondary and tertiary structure. Science 252, 1285–1288 (1991). 1656523 10.1126/science.1656523

[b44] Weiner M. P. *et al.* Site-directed mutagenesis of double-stranded DNA by the polymerase chain reaction. Gene 151, 119–123 (1994). 7828859 10.1016/0378-1119(94)90641-6

[b45] Volkov A. N., Vanwetswinkel S., Van de Water K. & van Nuland N. A. J. Redox-dependent conformational changes in eukaryotic cytochromes revealed by paramagnetic NMR spectroscopy. J. Biomol. NMR 52, 245–256 (2012). 22318343 10.1007/s10858-012-9607-8

[b46] Volkov A. N. & van Nuland N. A. J. Solution NMR study of the yeast cytochrome *c* peroxidase : cytochrome *c* interaction. J. Biomol. NMR 56, 255–263 (2013). 23708935 10.1007/s10858-013-9744-8

[b47] Delaglio F. *et al.* NMRPipe: a multidimensional spectral processing system based on UNIX pipes. J. Biomol. NMR 6, 277–293 (1995). 8520220 10.1007/BF00197809

[b48] Vranken W. F. *et al.* The CCPN data model for NMR spectroscopy: development of a software pipeline. Proteins 59, 687–696 (2005). 15815974 10.1002/prot.20449

[b49] Schwieters C. D., Kuszewski J. J., Tjandra N. & Clore G. M. The Xplor-NIH NMR molecular structure determination package. J. Magn. Reson. 160, 65–73 (2003). 12565051 10.1016/s1090-7807(02)00014-9

[b50] Schwieters C. D., Kuszewski J. J. & Clore G. M. Using Xplor-NIH for NMR molecular structure determination. Prog. Nucl. Magn. Reson. Spectrosc. 48, 47–62 (2006).

[b51] Iwahara J., Schwieters C. D. & Clore G. M. Ensemble approach for NMR structure refinement against ^1^H paramagnetic relaxation enhancement data arising from a flexible paramagnetic group attached to a macromolecule. J. Am. Chem. Soc. 126, 5879–5896 (2004). 15125681 10.1021/ja031580d

[b52] Summers F. E. & Erman J. E. Reduction of cytochrome *c* peroxidase compounds I and II by ferrocytochrome *c*. A stopped-flow kinetic investigation. J. Biol. Chem. 263, 14267–14275 (1988). 2844764

[b53] Marcus R. A. & Sutin N. Electron transfers in chemistry and biology. Biochim. Biophys. Acta 811, 265–322 (1985).

[b54] Rosales-Hernández M. C. *et al.* Theoretical study of heme derivatives under DFT calculations. J. Mol. Struct. 804, 81–88 (2007).

[b55] Liptak M. D., Wen X. & Bren K. L. NMR and DFT investigation of heme ruffling: functional implications for cytochrome *c*. J. Am. Chem. Soc. 132, 9753–9763 (2010). 20572664 10.1021/ja102098p PMC2914482

[b56] Balabin I. A., Hu X. & Beratan D. N. Exploring biological electron transfer pathway dynamics with the *Pathways* plugin for VMD. J. Comput. Chem. 33, 906–910 (2012). 22298319 10.1002/jcc.22927 PMC3288650

[b57] Baker N. A., Sept D., Joseph S., Holst M. J. & McCammon J. A. Electrostatics of nanosystems: application to microtubules and the ribosome. Proc. Natl Acad. Sci. USA 98, 10037–10041 (2001). 11517324 10.1073/pnas.181342398 PMC56910

[b58] Schwieters C. D. & Clore G. M. Reweighted atomic densities to represent ensembles of NMR structures. J. Biomol. NMR 23, 221–225 (2002). 12238594 10.1023/a:1019875223132

