# Supplementary material for: The low-affinity complex of cytochrome c and its peroxidase
Source: Nat Commun. 2015 May 6;6:7073. doi: 10.1038/ncomms8073 (PMC4432590; doi:10.1038/ncomms8073)
Supplement: Supplementary Information — Supplementary Figures 1-7, Supplementary Table 1, Supplementary Note 1 and Supplementary References [file ncomms8073-s1.pdf]

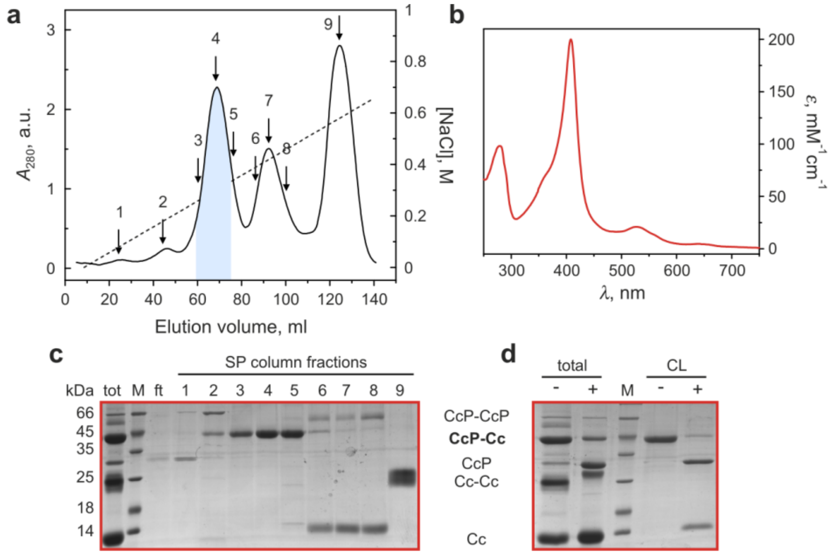

Supplementary Figure 1 | Characterization of the E290C CcP – K73C Cc disulfide CL. (**a**) Purification of the CL by cation-exchange chromatography. The solid and dashed lines show the UV absorbance and the linear salt gradient (from 0 to 1 M NaCl in 20 mM NaP_i pH 6.0), respectively, for protein elution from a HiTrap SP FF column (GE Healthcare). Protein fractions analyzed by SDS-PAGE are indicated by the arrows. The pooled fractions, containing the pure CL, are highlighted. (**b**) The UV-vis spectrum of the purified CL. (**c**-**d**) SDS-PAGE analysis of (**c**) the SP column fractions and (**d**) the CL samples before and after the purification. "M" denotes the molecular weight marker, with the values indicated on the left. (**c**) Non-reducing SDS-PAGE of the total reaction mixture before the purification ("tot"), the column flow-through ("ft"), and the protein fractions labeled in (**a**). (**d**) Samples of the CL reaction mixture ("total") and the CL stock after purification ("CL"), without (-) and with (+) 10 mM DTT.

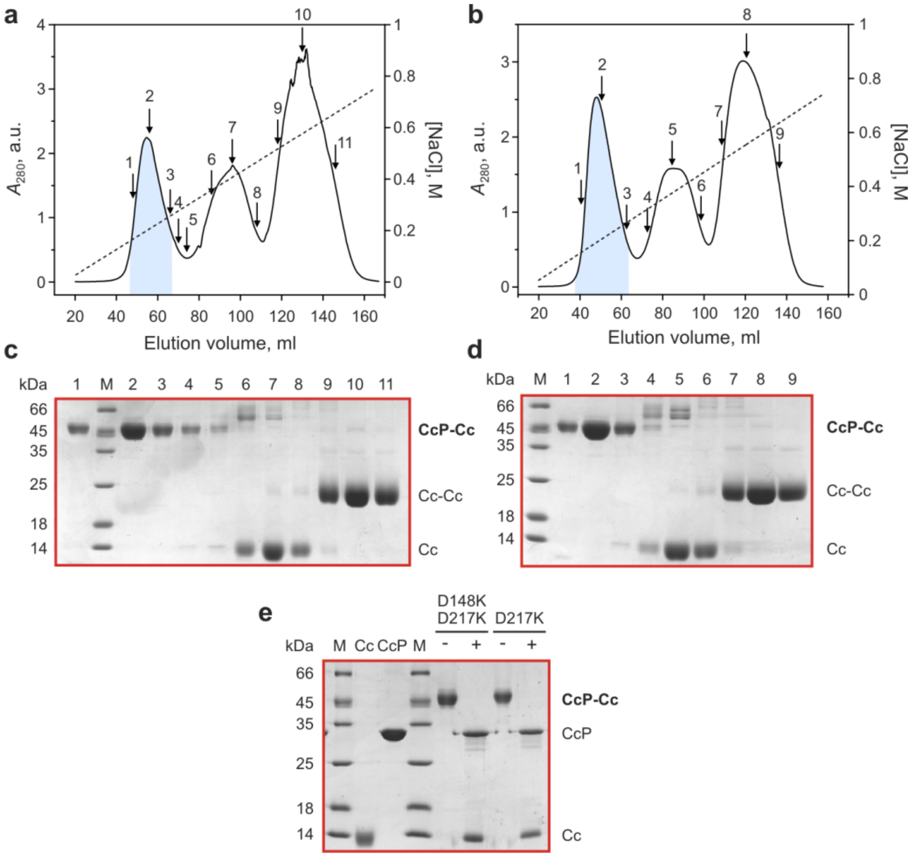

Supplementary Figure 2 | Characterization of the charge-reversal variants of V197C CcP – A81C Cc disulfide CL. Preparation of (**a**, **c**) D217K/V197C and (**b**, **d**) D148K/D217K/V197C [D, ^{13}C, ^{15}N] CcP – A81C Cc CLs. (**a**, **b**) Purification of the CLs by cation-exchange chromatography. The solid and dashed lines show the UV absorbance and the linear salt gradient (from 0 to 1 M NaCl in 20 mM NaP_i pH 6.0), respectively, for protein elution from a HiTrap SP FF column (GE Healthcare). Protein fractions analyzed by SDS-PAGE are indicated by the arrows. The pooled fractions, containing the pure CLs, are highlighted. (**c**, **d**) Non-reducing SDS-PAGE of the SP column fractions labeled in (**a**) and (**b**), respectively. "M" denotes the molecular weight marker, with the values indicated on the left. (**e**) SDS-PAGE of the freshly-purified CLs with (+) and without (-) the disulfide reducing agent DTT. The "+" samples contained 125 mM DTT and were incubated for 15 min at 99 °C prior to analysis. The wt proteins are included for reference.

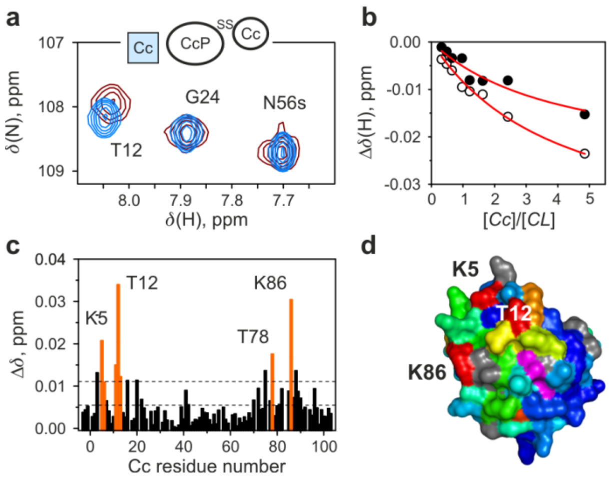

Supplementary Figure 3 | Analysis of the ^{15}N Cc binding to the E290C CcP – K73C Cc covalent CL. (**a**) A region of the overlaid [^{1}H, ^{15}N] HSQC spectra of the free ^{15}N Cc (blue) and in the presence of 5 molar equivalents of the CL (brown), showing typical chemical shift perturbations upon complex formation. The inset schematically depicts the experimental setup, with the NMR-active protein colored light blue. The symbol "s" identifies a sidechain amide resonance. (**b**) NMR chemical shift titrations of Cc HN resonances of K86 (filled circles) and K5 (open circles). The curves were fitted simultaneously to a binding model with the shared K_D. The solid lines show the best fits with the K_D value of 1.83 ± 0.78 mM. (**c**) Binding-induced, combined chemical shift perturbations ($\Delta\delta$) of the backbone amides of ^{15}N Cc. The horizontal lines indicate the average $\Delta\delta$ and the average plus one standard deviation. Several clusters of residues most affected by the binding are indicated by the labels, with the $\Delta\delta$ colored orange. (**d**) The $\Delta\delta$ heat map (colored from 0.001 ppm in blue to 0.02 ppm in red; prolines and the residues with unassigned or unobserved backbone amide resonances are in grey) of the Cc interface, with the heme group colored magenta. The labels indicate several residues affected by the binding. All experiments were conducted in 20 mM NaP_i pH 6.0 at 25 °C.

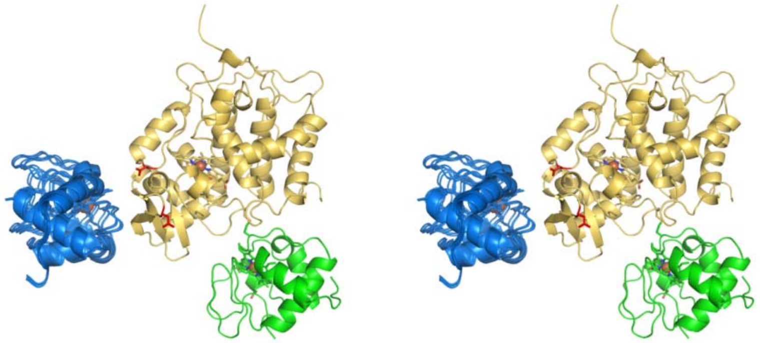

Supplementary Figure 4 | Dominant form of the CL-Cc complex. Stereo image showing fifteen lowest-energy structures of the CL-Cc complex, aligned over CL atoms. The positional pair-wise root-mean-square deviation of the Cα atoms of Cc from those in the mean structure is 1.40 ± 0.58 Å. Cc bound to the low affinity CcP site is shown in blue, while the cross-linked CcP and Cc are colored yellow and green, respectively. The CcP residues D148 and D217 are shown as red sticks.

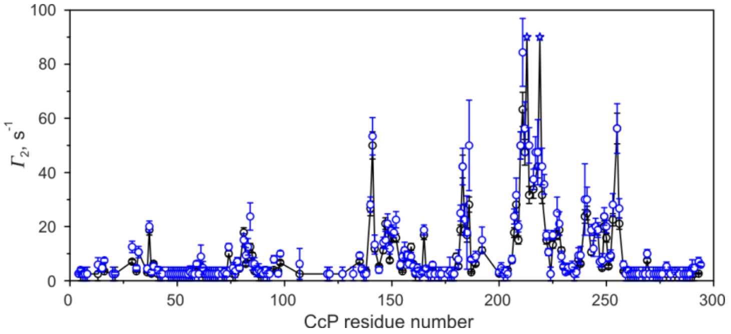

Supplementary Figure 5 | Reproducibility of the PRE measurements. The black and blue symbols refer to CcP-observed Γ_2 PREs for the CL in the presence of 3 molar equivalents of Cc E88C-EDTA(Mn^{2+}) in 20 mM NaP_i pH 6.0 for two separately prepared samples.

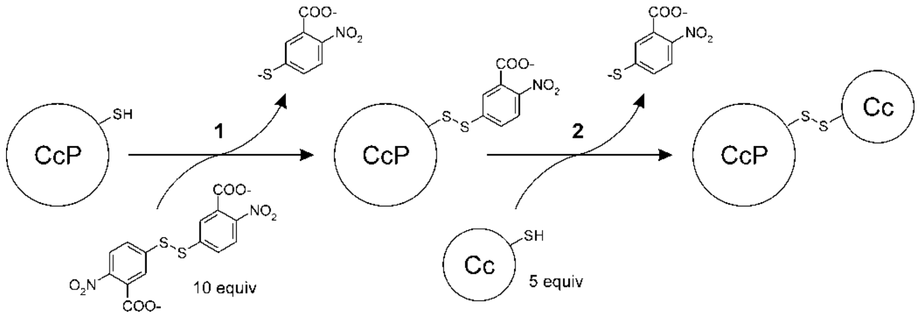

Supplementary Figure 6 | A reaction scheme of the synthesis of the covalent protein CLs used in this work. In the first step, a single-cysteine CcP variant is treated with the 10-fold molar excess of 5,5'-dithiobis-(2-nitrobenzoate) [DTNB or Ellman's reagent], yielding the modified protein and 3-thio-6-nitrobenzoate (TNB). In the second step, the modified CcP is reacted with the 5-fold excess of a single-cysteine Cc variant, producing the desired disulfide CL.

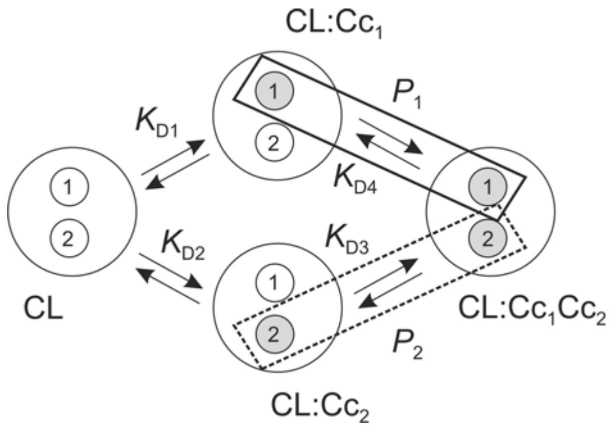

Supplementary Figure 7 | A model for Cc binding to two non-overlapping sites on CL. The occupancy of the two Cc sites, shown as numbered circles, is indicated by white (free) and gray (occupied). P_1 and P_2 are the total populations of CLs with Cc bound at the site 1 or 2, marked by the solid and dashed rectangles, respectively.

Supplementary Table 1 | Structural statistics for the CL-Cc complex.

	D50C	E66C	E88C	Total
Number of PRE restraints[a]				
Upper limit only[b]	172	190	149	511
Two bounds[c]	20	6	45	71
Total	192	196	194	582
	Dominant form		Entire complex	
Restraint violations				
Deviation from the nearest bound,[d] s^{-1}	6.31 ± 3.72		2.38 ± 2.75	
Deviation from the target,[e] s^{-1}	7.33 ± 3.99		3.64 ± 2.84	
Q factor[f]	0.66 ± 0.01		0.39 ± 0.02	
Molecular refinement quality				
Intermolecular repulsion energy,[g] kcal/mol	0.06 ± 0.08		0.09 ± 0.04	
Interfacial compactness energy,[h] kcal/mol	146.6 ± 10.2		459.2 ± 95.1	
	Cα atoms		Cα, N, C′, O atoms	
Coordinate precision,[i] Å	1.40 ± 0.58		1.41 ± 0.58	

[a] Given for three different Cc-EDTA(Mn^{2+}) attachment sites (Fig. 3a);

[b] $\Gamma_{2,obs} \leq 5$ s^{-1} restraints for the residues experiencing no PRE effects;

[c] $\Gamma_{2,obs} \pm \delta\Gamma_{2,obs}$ restraints for the residues experiencing intermolecular PREs;

[d] Calculated as $(\Gamma_{2,obs} - \delta\Gamma_{2,obs}) - \Gamma_{2,calc}$ or $\Gamma_{2,calc} - (\Gamma_{2,obs} + \delta\Gamma_{2,obs})$ for the lower- and upper-bound violations, respectively, for the residues in the lowest-energy CL-Cc solutions;

[e] Calculated as $|\Gamma_{2,obs} - \Gamma_{2,calc}|$ for the residues in the lowest-energy CL-Cc solutions;

[f] PRE Q factor (Equation 1) calculated for 15 lowest-energy CL-Cc solutions;

[g] Quartic repulsive term calculated for 15 lowest-energy CL-Cc solutions with the van der Waals force constant of 4 kcal·mol^{-1}·$Å^{-4}$ and van der Waals radius scaling factor of 0.8;

[h] Collapse energy term for the radius-of-gyration pseudopotential, used to ensure compactness of the calculated complexes, calculated for 15 lowest-energy CL-Cc solutions;

[i] Root-mean-square deviation of the atomic coordinates for 15 lowest-energy structures of the dominant CL-Cc form (Supplementary Fig. 4) from those in the mean structure.

Supplementary Note

Binding analysis of the low-affinity complex. The Cc-CL binding equilibrium can be described by the model of two non-overlapping binding sites, Supplementary Fig. 7 (also see text). The microscopic dissociation constants for the two sites, K_{D1} and K_{D2}, are given by the Supplementary Equation 1:

$$K_{\mathrm{D}1,2} = \frac{[CL][Cc]}{[CL:Cc]_{1,2}} = \frac{([CL]_{tot} - [CL:Cc]_1 - [CL:Cc]_2)([Cc]_{tot} - [CL:Cc]_1 - [CL:Cc]_2)}{[CL:Cc]_{1,2}} \quad (1),$$

where $[CL]$ and $[Cc]$ are the concentrations of the free proteins, $[CL]_{\mathrm{tot}}$ and $[Cc]_{\mathrm{tot}}$ are the total concentrations of CL and Cc in the sample, and $[CL{:}Cc]_1$ and $[CL{:}Cc]_2$ are the concentrations of CL with Cc bound at the site 1 and 2, respectively. The populations for the two binding sites are given by $P_{1,2}$ = $[CL{:}Cc]_{1,2}/[CL]_{\mathrm{tot}}$. Expressed in terms of the total protein concentration, C, for the equimolar solution of CL and Cc by substituting $[CL]_{\mathrm{tot}}$ = $[Cc]_{\mathrm{tot}}$ = C and $[CL{:}Cc]_{1,2}$ = $P_{1,2}[CL]_{\mathrm{tot}}$= $P_{1,2}C$ into the Supplementary Equation 1, the $K_{\mathrm{D}1}$ and $K_{\mathrm{D}2}$ are given by the Supplementary Equation 2:

$$K_{\mathrm{D}1,2} = \frac{(C - P_1C - P_2C)^2}{P_{1,2}C} = C\,\frac{(1 - P_1 - P_2)^2}{P_{1,2}} \quad (2)$$

Finally, the macroscopic dissociation constant (K_{Dtot}) can be obtained from the individual microscopic constants for the two binding sites, Supplementary Equation 3:[1]

$$K_{\mathrm{Dtot}} = \frac{K_{\mathrm{D}1}K_{\mathrm{D}2}}{K_{\mathrm{D}1}+K_{\mathrm{D}2}} \quad (3)$$

The error on the K_{Dtot} value, δK_{Dtot}, is propagated from the errors in $K_{\mathrm{D}1}$ and $K_{\mathrm{D}2}$, $\delta K_{\mathrm{D}1}$ and $\delta K_{\mathrm{D}2}$, Supplementary Equation 4:[2]

$$\delta K_{\mathrm{Dtot}} = \sqrt{\left(\frac{\partial K_{\mathrm{Dtot}}}{\partial K_{\mathrm{D}1}}\delta K_{\mathrm{D}1}\right)^2 + \left(\frac{\partial K_{\mathrm{Dtot}}}{\partial K_{\mathrm{D}2}}\delta K_{\mathrm{D}2}\right)^2} = \sqrt{\left[\left(\frac{K_{\mathrm{D}2}}{K_{\mathrm{D}1}+K_{\mathrm{D}2}}\right)^2 \delta K_{\mathrm{D}1}\right]^2 + \left[\left(\frac{K_{\mathrm{D}1}}{K_{\mathrm{D}1}+K_{\mathrm{D}2}}\right)^2 \delta K_{\mathrm{D}2}\right]^2} \quad (4)$$

As explained in the text, we experimentally determined $K_{\mathrm{D}1}$ = 2.0 ± 0.4 mM and established the relative populations of CLs with Cc bound at the site 1 or 2, p_1 = 1 and p_2 = 0.75 ± 0.15. Solving the Supplementary Equation 2 for the case of P_2 = (0.75 ± 0.15)P_1 and C = 0.5 mM, we obtained the populations of the two binding sites, P_1 = 14.2 ± 0.4 % and P_2 = 10.6 ± 1.5 %, and then calculated the other microscopic constant, $K_{\mathrm{D}2}$ = 2.7 ± 0.5 mM. Finally, the value of $K_{\mathrm{D,tot}}$ = 1.2 ± 0.2 mM was obtained from the Supplementary Equations 3 and 4.

The population of CL with Cc bound at both sites simultaneously can be found as follows. First, we note that the concentration of CL with Cc bound at the site 1 or 2 is the sum of the contributions from the respective binary and ternary complexes, $[CL{:}Cc]_{1,2}$ = $[CL{:}Cc_{1,2}]$ + $[CL{:}Cc_1{:}Cc_2]$ = $P_{1,2}C$. Then, we calculate the total concentration of the ternary complex as the sum of the contributions from the top- and bottom-right branches of the binding scheme in Supplementary Fig 7, $[CL{:}Cc_1{:}Cc_2]$= $[CL{:}Cc_1{:}Cc_2]_1$ + $[CL{:}Cc_1{:}Cc_2]_2$. Denoting the latter three terms as x, x_1, and x_2, respectively, we obtain the expression for $K_{\mathrm{D}3}$ and $K_{\mathrm{D}4}$ dissociation constants (defined in Supplementary Fig 7), Supplementary Equation 5:

$$K_{\mathrm{D}3,4} = \frac{[CL:Cc_{2,1}][Cc]}{[CL:Cc_1:Cc_2]_{2,1}} = \frac{(CP_{2,1} - x)(C - [CL:Cc_1] - [CL:Cc_2] - 2[CL:Cc_1:Cc_2])}{x_{2,1}} = C\,\frac{(CP_{2,1} - x)(1 - P_1 - P_2)}{x_{2,1}} \quad (5)$$

Taking x_1 and x_2 from the Supplementary Equation 5 and solving the equation for $x = x_1 + x_2$, we obtain the fraction of CL with Cc bound to both sites, Supplementary Equation 6:

$$\frac{x}{[CL]_{\text{tot}}} = \frac{x_1 + x_2}{C} = \frac{C(1 - P_1 - P_2)(P_1 K_{\text{D3}} + P_2 K_{\text{D4}})}{C(1 - P_1 - P_2)(K_{\text{D3}} + K_{\text{D4}}) + K_{\text{D3}} K_{\text{D4}}} \quad (6)$$

If the Cc interaction at one site does not influence that at the other (*i.e.* $K_{\text{D3}} = K_{\text{D1}}$ and $K_{\text{D4}} = K_{\text{D2}}$, Supplementary Fig 7), then 3.0 ± 0.3 % of CL has Cc bound at both sites simultaneously (Supplementary Equation 6). However, considering small separations between the Cc molecules bound at the two sites (main-text Fig. 5b), a strong electrostatic repulsion between them is expected to significantly impair, if not altogether abolish, the ternary complex formation. Thus, the value of 3.0 ± 0.3 % represents an upper limit for the fraction of CL with Cc bound to both sites.

Supplementary References

1. Nakani, S., Vitello, L. B. & Erman, J. E. Characterization of four covalently-linked yeast cytochrome *c* / cytochrome *c* peroxidase complexes: evidence for electrostatic interaction between bound cytochrome *c* molecules. *Biochemistry* **45**, 14371-14378 (2006).

2. Bevington, P. R. & Robinson, D. K. Data reduction and error analysis for the physical sciences. McGraw-Hill, Boston (2003).
